# CFD-driven neural network and SVM models for airfoil aerodynamic enhancement with bio-inspired riblets and plasma actuation

**DOI:** 10.1038/s41598-026-39525-0

**Published:** 2026-03-19

**Authors:** Kailam Vijayakumar Karthikeyan, Amritha Raju, Jake Jain, Srinivasan Lekshmanan, Harish Rajan

**Affiliations:** School of Mechanical Engineering, Vellore Institute of Technology, Chennai, Tamil Nadu 600127 India

**Keywords:** Bio-inspired riblets, Artificial neural network, Scaled conjugate gradient, Support vector machine, Aerodynamic performance, CFD-machine learning integration, Engineering, Mathematics and computing

## Abstract

Enhancing the airfoil performance using advanced flow control techniques is an important challenge in the aerospace and automotive industries. In this article a hybrid computational and artificial intelligence (AI) approach is developed for aerodynamic performance enhancement of a NACA4412 airfoil by using a combination of bio inspired riblets and active plasma control. Semi circular, triangular and fillet riblets were tested with alternating current dielectric barrier discharge (AC-DBD) plasma devices and simulations were performed using computational fluid dynamics (CFD) technique at a Reynolds number of $3.1\times 10^6$ with angles of attack ranging from $0^\circ$ to $20^\circ$. The performance assessment parameters considered in this work include lift coefficient $(C_L)$, drag coefficient $(C_D)$, lift to drag ratio ($C_L/C_D$) ratio and vorticity contours in order to infer flow stability and efficiency. The simulation results indicated a reduced drag coefficient using riblets due to modifications in wall boundary layers along with a subsequent addition of AC-DBD devices to suppress vortex formation, delay separation and promote higher lift. Among all designs the bio-inspired fillet riblet with plasma actuator recorded maximum improvements with respect to $C_L$ with an increase of $20.38\%$ and $45.71\%$ in $C_L/C_D$ ratio with respect to base configuration. Additionally, to facilitate model prediction and simulation the machine learning algorithms were established using artificial neural networks with scaled conjugate gradient learning algorithm and support vector machine regressions. The results obtained indicated excellent model accuracy with high correlation coefficient and low mean squared error measurements using $C_L$ and $C_D$ predictions. These findings highlight the dual role of CFD and AI in the optimization of flow control strategies and confirm that the integration of physics based simulations with machine learning offers an efficient and reliable pathway for prediction and design innovation in aerodynamics.

## Introduction

The growing complexity of modern aerodynamic systems and the increasing demand for fast and accurate predictive tools have driven significant interest in coupling computational fluid dynamics (CFD) with machine learning (ML) techniques. CFD allows for detailed physics based understanding of flow behaviour but takes a lot of computational resources and time particularly when performing parametric studies across extensive ranges of operation. On the other hand machine learning methods trained with CFD or experimental data can produce suitably accurate and rapid aerodynamic coefficients and flow characteristics for effective design exploration and optimization. Such CFD-ML platforms are becoming more widely accepted as strong tools for bridging high fidelity simulation with everyday engineering practice. As there is a growing focus on fuel efficiency and performance the flow control measures have become of greater interest^1^. These techniques tend to be segregated as passive and active, each with its own benefits. Passive techniques which do not involve any input of external energy, are vortex generators^2^, riblets^3^, dimples^4^ cavities^5^, slots^6^ and gurney flaps^7^. Among them are riblets which are microscale grooves oriented parallel to the flow that reduce drag coefficients ($C_D$) in turbulent boundary layers very effectively. By inhibiting spanwise vortex motion the riblets minimize skin friction drag with little structural change. Drawn from shark skin micro structures the riblets provide a bio-inspired solution that unites natural flow control regimes with pragmatic engineering applications which providing superior aerodynamic effectiveness without radical redesigns or actuators^8^.

Riblets have been widely employed in aerospace, marine and energy systems. Applications include aircraft wings^9^, fuselages^10^, engine nacelles^11^ and turbo machinery blades^12^ where they minimize frictional losses and improve fuel economy. In marine engineering, riblets applied to ship hulls^13^ and underwater vehicles^14^ reduce turbulent drag thereby enhancing range and efficiency. Additionally, riblets improve energy capture in turbulent conditions by reducing drag on wind turbine blades^15^. Riblets are strategically positioned on airfoils to improve overall aerodynamic performance, delay separation and smooth airflow. Lu et al.^16^ proposed a bio-inspired adaptive drag reduction surface combining transverse grooves, flexible deformation and hydrophobic coatings, achieving 28% $C_D$ reduction in marine environments. Similarly, Harun et al.^17^ experimentally studied converging-diverging riblets on a flat-bottomed airfoil bump under adverse pressure gradients. The results stated that the riblet geometry significantly modifies boundary layer development and turbulence structures with diverging riblets providing measurable drag reduction.

Additional riblet extensions involve bio-inspired herringbone grooves on NACA0012 airfoils with optimized depth and yaw angles found to mitigate separation and increase the stall margin^18^. Experiments with unmanned aerial vehicles (UAV) showed that trapezoidal riblets decreased $C_D$ by 7.5%, while blade-shaped riblets had little effect if not optimally spaced^19^. Likewise, trapezoidal riblets on NACA0015 yielded a maximum drag reduction of 21% with particle image velocimetry visualization confirming enhanced momentum transfer and reduced wake size^20^. Zigzag riblets have also been shown to attenuate turbulence intensities more effectively than conventional streamwise riblets^21^. In addition, triangular riblets combined with spanwise wall jet forcing achieved up to 20% drag reduction, far surpassing riblets alone^22^. Large eddy simulations further revealed the influence of riblet geometry, comparing square and semi-circular designs, on vortex formation, transition and flow instabilities^23^. Similarly, Sharma et al.^24^ numerically demonstrated that shark skin inspired riblets reduce near wall turbulence and achieved up to 9.46% drag reduction on flat plates. Collectively, the literature shows that riblets passively enhance aerodynamics by modulating near wall flow, delaying separation and reducing drag. However, their benefits are modest with limited lift coefficient ($C_L$) improvement, sensitivity to operating conditions and manufacturing challenges on curved or large surfaces restricting widespread implementation in aerospace and turbo machinery.

Unlike passive methods, active flow control can adapt to changing conditions through techniques such as plasma actuators^25–27^, synthetic jets^28,29^ and suction/blowing systems^30,31^. Among them, dielectric barrier discharge (DBD) plasma actuators are particularly attractive due to their fast response, lightweight design and absence of moving parts. They provide real time boundary layer control with proven effectiveness in delaying separation, reducing drag and enhancing lift across UAV, rotorcraft^32,33^ and wind turbines^34,35^. The effectiveness of plasma actuators has been validated in diverse configurations. Zuo et al.^36^ reported a 21.8% improvement in lift to drag ratio ($C_L/C_D$) on a two element airfoil due to reduced separation. Spanwise plasma arrays achieved 40% drag reduction at low Reynolds numbers by eliminating laminar separation bubbles^37^. Optimal actuator placement at the leading edge maximized efficiency in high Reynolds flows, though excessive voltages increased drag^38^. Integrated experimental, numerical and ML based studies achieved lift and $C_L/C_D$ gains of 65% and 161%, respectively^39^. Additional contributions include shock boundary layer interaction control in transonic flows^40^, torque reduction of 21.6% on rotor blades^41^ and validated Suzen model predictions with 7.2% lift gain on the RAE2822 airfoil^42^. Furthermore, trailing-edge plasma actuators increased post stall lift by 17% on a NACA0015 with unsteady actuation showing superior performance^43^. Collectively, these results confirm that plasma actuators actively modulate boundary layers, delay separation and enhance aerodynamic performance in both low and high speed flows.

In recent years, machine learning has been shown to be a strong supplement to CFD and experimental approaches to aerodynamic prediction and optimization. Mohssen et al.^44^ used artificial neural networks (ANN) with quadratic fitting to forecast $C_L$ and $C_D$ over Mach numbers, exhibiting strong generalization to novel airfoils. Deep learning architectures incorporating attention have been employed to reconstruct flow fields three times as quickly as traditional CFD^45^. Hybrid CFD and ANN-genetic algorithm methods optimized UAV airfoils to closely match CFD predictions while selecting designs with desirable stall behavior^46^. Comparative analyses indicated that feed-forward back propagation neural networks compare favorably with multiple linear regression for aerodynamic coefficient prediction^47^. Reinforcement learning has also been adopted for intelligent flow control, effectively suppressing separation at high angles of attack (AOA) through vorticity based reward functions^48^. Contrastive learning has recently been combined with support vector machines (SVM) through novel multi view deep SVM models employing discriminative contrastive loss for multi class classification. This approach enhances inter view diversity and cross view consistency using adaptive view weighting and similarity constraints, achieving superior accuracy compared to state-of-the-art methods^49^. More recently, regression trees, support vector machines and back propagation neural networks (BPNN) have been evaluated with BPNN achieving the lowest mean squared error (MSE) for $C_L$ predictions and fine regression trees providing the best accuracy for $C_D$^50^.

This growing body of literature underscores the potential of combining physics-based CFD with artificial intelligence driven predictive modeling to deliver efficient, accurate and generalizable aerodynamic performance enhancement. Despite these advances, few studies have addressed the integrated use of bio-inspired riblets, plasma actuators and machine learning frameworks for airfoil optimization at high Reynolds numbers. The present work develops a CFD-ML framework to assess and predict the aerodynamic impact of hybrid riblet and plasma flow control strategies on a NACA4412 airfoil. Specifically, bio-inspired riblet geometries are integrated with AC-DBD plasma actuation, an approach that remains largely unexplored. While riblets passively reduce skin friction drag at lower angles of attack, they often increase $C_D$ and alter $C_L$ at higher angles due to premature flow separation. In contrast, plasma actuators delay separation but suffer from localized influence and energy costs. By combining these techniques this study demonstrates a synergistic effect with the bio-inspired fillet riblet coupled with plasma actuation achieving superior lift enhancement, stall delay and improvements in the $C_L/C_D$ ratio compared to either method alone. A further novelty lies in coupling high fidelity CFD simulations with machine learning models, namely artificial neural networks trained using the scaled conjugate gradient (SCG) algorithm and support vector machine regression. Both models yielded highly accurate predictions of $C_L$ and $C_D$ with minimal error by confirming the reliability of the CFD-ML approach. Overall, this integrated framework offers an efficient pathway for aerodynamic optimization by reducing the need for exhaustive testing while supporting the advancement of hybrid flow control systems that integrate bio-inspired passive features with active plasma actuation.

## Methodology

### Computational domain

**Fig. 1:**
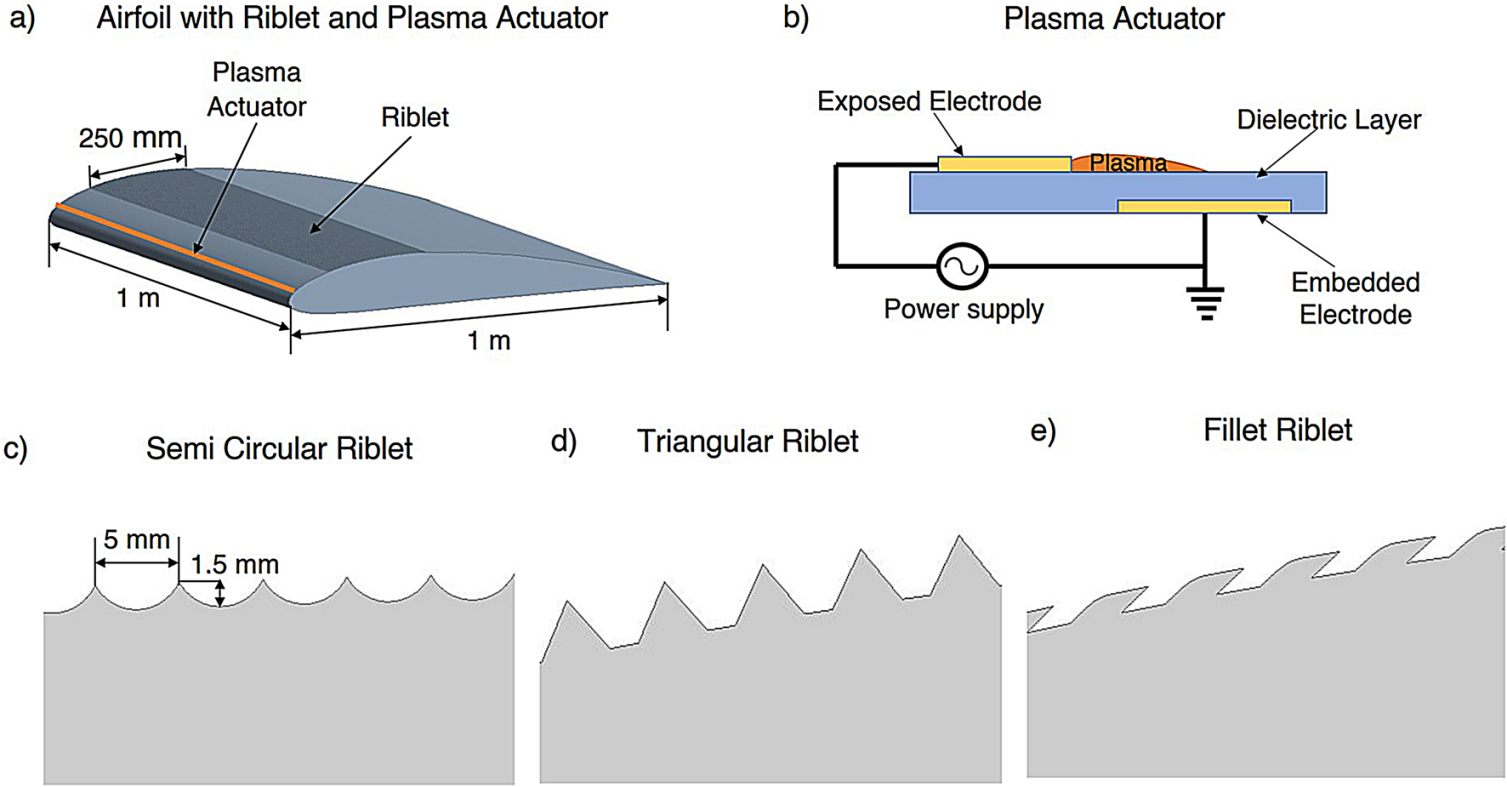
Schematic diagram of NACA4412 airfoil fitted with a riblet and plasma actuator.

This study investigates an unsymmetrical NACA4412 airfoil with a chord length of 1 m which modified by introducing riblets on the suction side at 25% of the chord length from the leading edge. Three riblet geometries were analyzed: semi-circular riblets (SCR), triangular riblets (TR) and bio inspired fillet riblets (FR). Additionally, a dielectric barrier discharge plasma actuator (PA) were installed near the leading edge at 2% of the chord length. The plasma actuator is positioned at $2\%$ of chord because the boundary layer near the leading edge is extremely thin and highly receptive to external forcing. Introducing body force at this early stage stabilizes the developing shear layer and energizes the near-wall flow and delays separation. Previous studies^41,51^ on airfoils indicate that actuator placement within 1 - 4% of chord provides the greatest separation control and lift enhancement. Thus selecting $2\%$ of chord ensures maximum control authority and consistency with established flow control. The geometric configurations of the riblets and actuator placement are illustrated in Fig. 1 where Fig. 1a shows the airfoil with riblets and actuator position and Fig. 1c–e, depict the riblet geometries. The riblets were designed with a width of 5 mm and a depth of 1.5 mm. Numerical simulations were performed for AOA ($\alpha$) from $0^{\circ }$ to $20^{\circ }$.

The riblet band is positioned starting at 25% of chord because the boundary layer over the NACA4412 becomes fully turbulent near this location and riblets are effective only in the turbulent regime. Extending the riblets over a length of 0.25 chord ensures coverage of the mid-chord region where drag is higher. Beyond 50% of chord the boundary layer thickens and the effectiveness of riblets diminishes which makes the chosen placement optimal for maximizing drag reduction^52–54^. The ACDBD plasma actuator consists of a 6 mm wide exposed electrode and a 10 mm wide embedded electrode which separated by a dielectric layer of total thickness 0.46 mm. The electrodes have a thickness of 0.05 mm while the embedded electrode is fully covered by the dielectric material^55^ as shown in Fig. 1b. A plasma region forms at the upstream edge of the exposed electrode which enables effective boundary layer momentum addition. The spanwise length of the actuator is set to 1 m.

The selection of riblet dimensions in the present study width $d = 5~\text {mm}$ and height $h = 1.5~\text {mm}$ is based on the expected boundary layer characteristics of the NACA4412 airfoil at a chord-based Reynolds number of $Re = 3.1 \times 10^{6}$. At such high Reynolds numbers, the turbulent boundary layer thickness near the mid-chord region decreases significantly which correspondingly shifts the viscous sublayer and buffer layer closer to the wall. For riblets to provide meaningful drag-reduction and separation-control benefits, their height must lie within the buffer layer where they can suppress spanwise motions of near-wall vortices without protruding into the log region^52–54^. Based on airfoil turbulent boundary layers at Reynolds number of $10^{6}$, the local boundary layer thickness on the suction surface of airfoil implying an optimal riblet height of approximately $10{-}20\%$ of the local boundary layer thickness consistent with the chosen value of $h = 1.5~\text {mm}$ and d = 5 mm of all three riblet cases^53,54^.

**Fig. 2:**
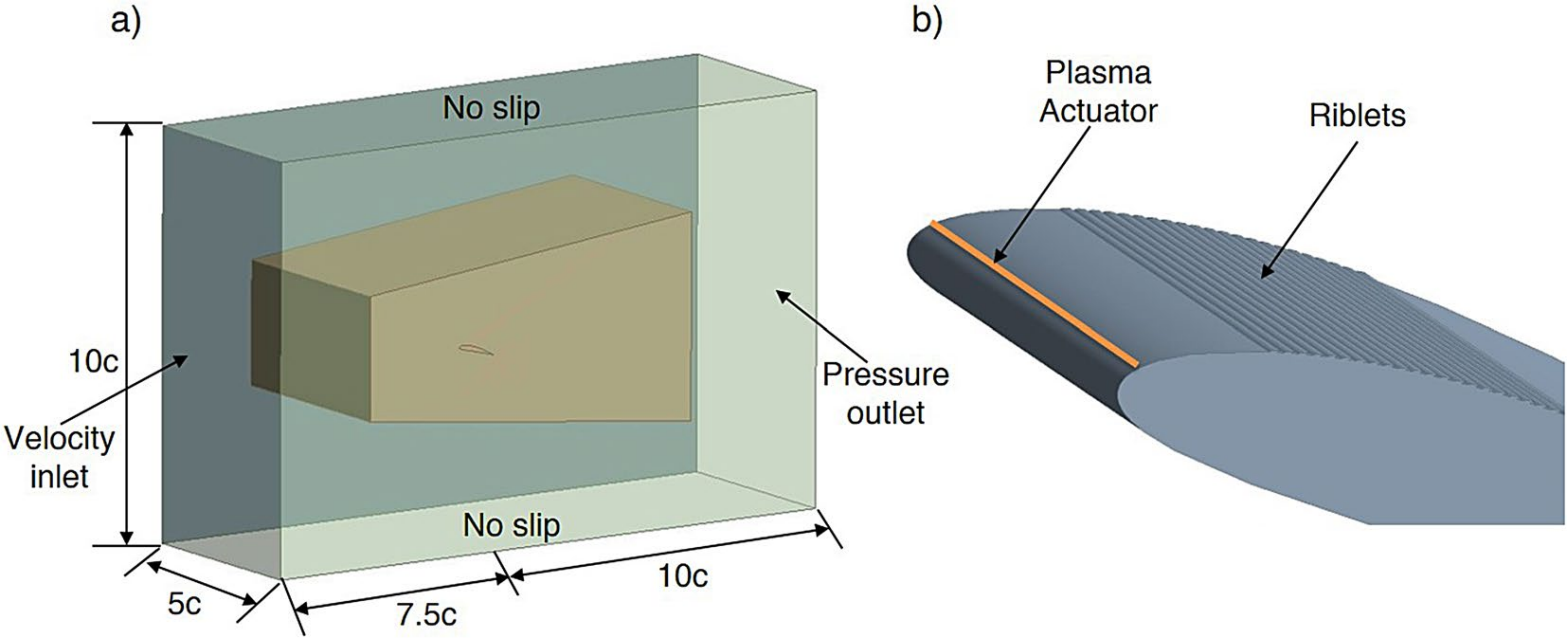
**a** Computational domain around airfoil, **b** 3D airfoil with riblets and plasma actuator.

For accuracy, the computational domain was constructed with boundary conditions^56^. Figure 2 illustrates the computational setup and the surface-mounted flow control devices used in the study. In Fig. 2a, the airfoil is placed inside a rectangular domain where the velocity inlet is positioned 7.5 chord upstream of the leading edge while the pressure outlet is located 10 chord downstream of its trailing edge while the top and lower far-field boundaries are positioned 5 chord lengths from the airfoil surfaces. A no-slip condition is applied on side, top and bottom walls to accurately resolve near-wall viscous effects^57,58^. In addition, the spanwise extent of the rectangular domain is set to 5 chord length to ensure sufficient spanwise flow development and to avoid any artificial confinement effects^57,58^. Figure 2b presents a three-dimensional view of the airfoil surface, highlighting the integration of the plasma actuator and riblet texture. The plasma actuator shown as a thin strip near the leading region of the upper surface which represents the exposed electrode used for producing the body force field while the riblets are arranged in the streamwise direction and extruded spanwise to form a textured band located downstream of the actuator. Together, these visualizations depict the domain configuration and the combined passive and active flow control arrangement investigated in this study.

#### Mathematical model

In this study, the airflow over the NACA4412 airfoil is modeled as three-dimensional, unsteady and incompressible turbulent flow with constant density. The governing equations are the Unsteady Reynolds-averaged Navier-Stokes (URANS) equations which capture the mean flow field along with turbulence effects^59^.

Continuity equation:

1 $$\begin{aligned} \frac{\partial u_i}{\partial x_i} = 0 \end{aligned}$$

Momentum equation:

2 $$\begin{aligned} \rho \frac{\partial u_i}{\partial t} + \rho u_j \frac{\partial u_i}{\partial x_j} = -\frac{\partial p}{\partial x_i} + \mu \frac{\partial }{\partial x_j} \left( \frac{\partial u_i}{\partial x_j} + \frac{\partial u_j}{\partial x_i} \right) + \frac{\partial }{\partial x_j} \left( -\rho \overline{u_i' u_j'} \right) + F_{i} \end{aligned}$$

In this context, *p* denotes pressure, $\mu$ represents dynamic viscosity, $\rho$ signifies fluid density, *t* indicates time, $F_{i}$ corresponds to the body force term and $-\rho \overline{u_i' u_j'}$ represents the Reynolds stress.

To model the impact of turbulence and flow separation, the Shear Stress Transport (SST) *k*-$\omega$ formulation was used. This formulation combines the near-wall robustness of the *k*-$\omega$ treatment with the free-stream independence of *k*-$\varepsilon$ model which is well suited to predict unfavorable pressure gradients and stall behavior of airfoils. Transport equations for turbulent kinetic energy (*k*) and specific rate of dissipation ($\omega$) are provided by^59,60^

3 $$\begin{aligned} & \frac{\partial k}{\partial t} + U_i \frac{\partial k}{\partial x_i} = \frac{\partial }{\partial x_i} \left[ \left( \nu + \sigma _k \nu _t \right) \frac{\partial k}{\partial x_i} \right] + P_k - C_\mu \omega k \end{aligned}$$

4 $$\begin{aligned} & \frac{\partial \omega }{\partial t} + U_i \frac{\partial \omega }{\partial x_i} = \frac{\partial }{\partial x_i} \left[ \left( \nu + \sigma _\omega \nu _t \right) \frac{\partial \omega }{\partial x_i} \right] + \gamma \frac{\omega }{k} P_k - \beta \omega ^2 + (1 - F_1) \frac{2 \sigma _\omega }{\omega } \frac{\partial k}{\partial x_i} \frac{\partial \omega }{\partial x_i} \end{aligned}$$

A production limiter is incorporated into the turbulence model to prevent excessive turbulence creation close to stagnation regions. As a result the production phrase is defined as^61^

5 $$\begin{aligned} & \widetilde{P}_k = \min \left( P_k,\, 20 \cdot \beta ^* \rho k \omega \right) , \end{aligned}$$

The calculation of eddy viscosity is performed as follows

6 $$\begin{aligned} & \mu _t = \frac{\alpha _1 k}{\max (\alpha _1 \omega , \Omega F_2)} \end{aligned}$$

where,

7 $$\begin{aligned} & \Omega = \left( \frac{2}{3} \overline{S}_{ij} \overline{S}_{ij} \right) ^{1/2}, \quad \overline{S}_{ij} = \frac{1}{2} \left( \frac{\partial \overline{U}_i}{\partial x_j} + \frac{\partial \overline{U}_j}{\partial x_i} \right) \end{aligned}$$

#### Plasma model

Plasma actuators are recognized for generating a body force within the flow by effectively introducing momentum into a localized region typically where the plasma is produced. When plasma actuator are incorporated into flow models, a body force term $F_{i}$ is introduced to the governing momentum equations. An empirical model to explain this effect was proposed by Shyy et al.^62^, offering a deeper understanding of the interaction mechanism between the plasma and the adjacent fluid flow. This modeling approach concentrates on representing how plasma induced body forces affect the flow. The electric field strength produced by the plasma is determined by

8 $$\begin{aligned} |\overrightarrow{E}|=\ E_0-k_1x-k_2y \end{aligned}$$

E${}_{0}$ represents the peak electric field strength were the constants k${}_{1}$ and k${}_{2}$ are determined to satisfy the breakdown threshold condition^62^. Under this formulation the body force term in Eq. (2) is expressed as:

9 $$\begin{aligned} & F_{i}= \overrightarrow{F} \end{aligned}$$

10 $$\begin{aligned} & \overrightarrow{F}=\vartheta {\rho }_ce_c\Delta t \overrightarrow{E} \end{aligned}$$

11 $$\begin{aligned} & \overrightarrow{E}=\left( \frac{|\overrightarrow{E}|k_2}{\sqrt{k^2_1+k^2_2}},\ \frac{|\overrightarrow{E}|k_1}{\sqrt{k^2_1+k^2_2}}, 0\ \right) \end{aligned}$$

Here, $\rho$ ${}_{c}$ denotes the electron charge density, $e_c$ is the elementary charge, $\vartheta$ represents the frequency of the applied voltage and $\Delta t$ is the discharge time. The parameter values used in the above equation are adopted from^62^. The terms in Eq. (11) are employed to adjust both the magnitude and spatial distribution of the body force. This approximation is considered sufficient to capture the essential physics of a plasma actuator system, as applied in the present study.

The mathematical formulation of the plasma model is discussed and the ACDBD plasma actuator is operated at a voltage of 5.67 kV and a driving frequency of 5 kHz using a sinusoidal waveform^63^. The actuator employs a standard asymmetric DBD arrangement consisting of an exposed electrode on the airfoil surface and a covered electrode embedded beneath the dielectric layer, as illustrated in Fig. 1b. The resulting body-force field is incorporated into the CFD solver through a user defined function (UDF) where the plasma forcing is applied as a steady source term and updated at each solver iteration to ensure proper coupling with the computed flow field. The UDF for implementing the plasma body force was developed in the C programming language, enabling efficient numerical integration into the solver.

### Artificial neural network model

Artificial neural networks are computational models inspired by the human brain, capable of learning nonlinear relationships from data. Unlike traditional methods that depend on explicit equations, ANN adapt through training, making them highly effective for complex and multi input multi output systems. They often surpass regression models in predictive accuracy and provide fast, reliable predictions without predefined formulations which makes them valuable across scientific and engineering applications^64,65^.

**Fig. 3:**
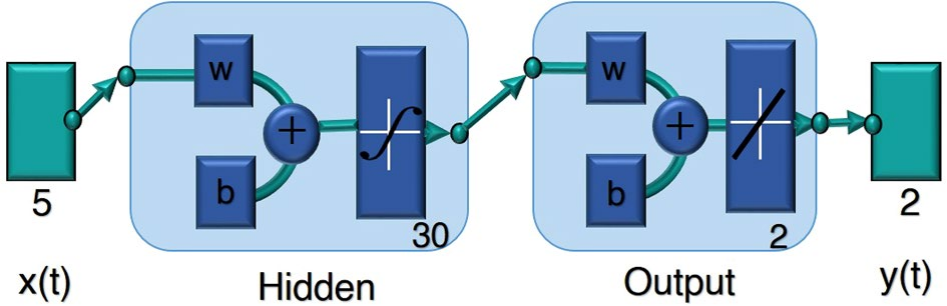
Schematic overview of the ANN model showing the input, hidden and output layers for aerodynamic performance estimation.

Let the input vector be represented by *x*(*t*) and the corresponding output vector by *y*(*t*). The network operates through weighted interconnections *w* and bias terms *b* across its layers. A standard ANN is composed of an input layer, one or more hidden layers and an output layer. Each hidden layer contains neurons that perform mathematical transformations and transmit the results forward. The ANN architecture used in this study is shown in Fig. 3 with the mapping between input, hidden and output layers defined by Eqs. (12) and (13)^66^.

12 $$\begin{aligned} & \beta _k = \rho _n \left( \sum _{j=0}^{h} \omega ^{(n)} \sigma _j \cdot \tau _j \right) \end{aligned}$$

13 $$\begin{aligned} & \tau _j = \rho _{n-1} \left( \sum _{i=0}^{N} \omega ^{(n-1)}_{ji} \mu _i \right) \end{aligned}$$

The number of neurons in the input is indicated by *n* in the equations above while the hidden and output layers are represented by *h* and *k*, respectively. The weighted activation in the hidden layer is represented by the quantity $\omega ^{(n)} \sigma$ and the activation function used at the output layer is indicated by $\beta _k$. The ANN model were implemented in MATLAB utilizing input datasets generated from CFD simulations. ANN model was developed with a single hidden layer comprising 30 neurons which structured to effectively learn the nonlinear relationship between the aerodynamic input parameters and their corresponding outputs. The rectified linear unit (ReLU) activation function was incorporated into the hidden layer to improve learning efficiency and quicker convergence. Meanwhile the output layer adopted a linear activation function which enables precise prediction of continuous aerodynamic coefficients such as lift and drag.

The SCG algorithm is a supervised learning approach used to train feed-forward neural networks. As a second order optimization technique it combines the efficiency of first-order procedures and the accuracy of conjugate gradient methods. By employing a step size scaling mechanism, SCG avoids line searches and is effective for large scale training. Its weight updates rely on both current and previous gradients, enabling faster convergence than standard gradient descent^66^. Equations (14) and (15) define the training data set and parameter vector formulation used in this framework.

14 $$\begin{aligned} & S_{r+1} = G_{r+1} + S_r \cdot \alpha _x \end{aligned}$$

15 $$\begin{aligned} & W_{r+1}= W_r + S_r \cdot \beta x \quad ; \quad r = 1, 2, 3, \dots \end{aligned}$$

In this case $S_r$ stands for the initial search direction vector, $W_r$ for the initial set of model parameters, $\alpha$ for the SCG scaling coefficient and $\beta$ for the learning rate.

#### Support vector machine

The support vector machine is a supervised learning algorithm commonly used for both classification and regression tasks. It determines the maximum margin hyperplane using support vectors and employs kernel functions to manage nonlinear data by mapping it to higher dimensional spaces. In regression tasks, support vector regression (SVR) predicts continuous outcomes via a regularized risk function. Unlike ANN which may converge to local minima, SVM uses convex optimization, guaranteeing a globally optimal solution while effectively handling nonlinearity and high dimensional data^50,67^.

16 $$\begin{aligned} f(x) = \omega \varphi (x) + b \end{aligned}$$

The function $\varphi (x)$ maps the input vector *x* into a higher-dimensional feature space. The parameter $\omega$ denotes the weight vector and $b$ is the bias term. The SVR model is trained by minimizing the regularized risk function, given as^67^

17 $$\begin{aligned} R(C) = C \cdot \frac{1}{n} \sum _{i=1}^{n} L(d_i, y_i) + \frac{1}{2} \Vert \omega \Vert ^2 \end{aligned}$$

Here, $C$ is the regularization parameter, $d_i$ is the target value for the $i^{\text {th}}$ observation and $n$ is the total number of observations. The term $\frac{1}{n} \sum _{i=1}^{n} L(d_i, y_i)$ corresponds to the empirical loss and the second term $\frac{1}{2} \Vert \omega \Vert ^2$ imposes a penalty on model complexity.

The loss function $L_\varepsilon (d, y)$, often referred to as the $\varepsilon$ and insensitive loss function is defined as^67^

18 $$\begin{aligned} L_\varepsilon (d, y) = {\left\{ \begin{array}{ll} |d - y| - \varepsilon & \text {if } |d - y| \ge \varepsilon \\ 0 & \text {otherwise} \end{array}\right. } \end{aligned}$$

In this context, $\varepsilon$ defines the width of the tube within which no penalty is given to errors. The regularization term $\frac{1}{2} \Vert \omega \Vert ^2$ ensures smoothness and generalization of the model. Upon applying the method of Lagrange multipliers and satisfying the Karush-Kuhn-Tucker conditions, the final decision function can be expressed in its dual representation as^67^

19 $$\begin{aligned} f(x, a_i, a_i^*) = \sum _{i=1}^{n} (a_i - a_i^*) K(x, x_i) + b \end{aligned}$$

Here, $a_i$ and $a_i^*$ are the Lagrange multipliers and $K(x, x_i)$ is the kernel function that computes the inner product in the transformed feature space, enabling the model to learn nonlinear patterns. In this study, the radial basis function (RBF) kernel a popular nonlinear kernel was selected due to its superior performance in estimating $H$ compared to other kernel types ^68^. The RBF kernel is mathematically defined as^67^

20 $$\begin{aligned} K_{\text {rbf}}(x, x_i) = \exp \left( \frac{-(x - x_i)^2}{2\sigma ^2} \right) \end{aligned}$$

Support vector machine is a widely used machine learning method that formulates classification as a convex optimization problem, identifying the hyperplane that maximizes class separation. It offers strong generalization, robustness to outliers and ease of model updating but can be computationally expensive and sensitive to high dimensionality, large datasets and imbalanced data ^69^.

#### Feature selection and normalization

The input variables were chosen in accordance with core aerodynamic considerations which reflects their direct influence on aerodynamic coefficients, boundary layer development and flow separation behavior around the airfoil. The key governing parameters are namely, the angle of attack, Reynolds number, plasma actuator frequency, plasma actuator location and total width of riblets play a crucial role in determining $C_L$ and $C_D$ generation by influencing separation of flow control and circulation strength. The output parameters comprised the $C_L$ and $C_D$ coefficients derived from validated CFD simulations. Together these data formed a supervised learning dataset used to train the neural network. In order to make the optimization process numerically stable and have all parameters contribute equally to the optimization the input features and output features were first normalized. The method of normalization used for each continuous variable *x* was the min-max normalization technique. Min-Max normalization transforms continuous variables to give a range of [0, 1]^70,71^.

21 $$\begin{aligned} x' = \frac{(x - min)(max_n - min_n)}{max - min} + min_n \end{aligned}$$

The true data value in this normalization strategy is represented by *x* while the dataset minimum and maximum values are indicated by $\text {min}$ and $\text {max}$, respectively. The desired range limits are specified by the parameters $\text {min}_n$ and $\text {max}_n$ and the final normalized value is denoted by $x'$. This process preserves the relative distribution of the data points while normalizing all variables to a consistent range between 0 and 1. The procedure of normalizing data by ensuring that data is scaled uniformly which improves numerical conditioning for stable convergence in training. Normalization factors were systematically recorded and consistently utilized throughout all phases of training, validation and testing by applying uniformity to data thereby enabling accurate denormalization of predicted aerodynamic coefficients. Thus a normalization step enhances stability of convergence performance of ANN which also improves predictive capability of ANN and provides greater generalization for ANN models under a broader spectrum of aerodynamic scenarios.

The performance of all the algorithms was evaluated to find the best approach for best prediction. Model performance was determined using common statistical measures such as mean squared error and regression coefficient (*R*). Strong prediction accuracy and good model performance were shown with a high *R* value and low MSE^50^.

22 $$\begin{aligned} & R = \sqrt{1 - \frac{\sum (M_i - P_i)^2}{\sum (M_i - \overline{M_l})^2}} \end{aligned}$$

23 $$\begin{aligned} & \textrm{MSE} = \frac{1}{n} \sum _{i=1}^{n} |P_i - M_i|^2 \end{aligned}$$

In this context, *n* represents the total number of data points while $P_i$ and $M_i$ denote the predicted and actual values, respectively. The symbol $\overline{M}_l$ corresponds to the mean of the measured values^50^.

### Boundary conditions and numerical methods

The turbulence quantities namely the turbulent kinetic energy (*k*) and the specific dissipation rate ($\omega$) were initialized using standard empirical correlations^72,73^.

24 $$\begin{aligned} & k = \frac{3}{2} (U_{\infty } I)^2 \end{aligned}$$

25 $$\begin{aligned} & \quad \omega = \frac{k^{1/2}}{l} \end{aligned}$$

where *I* represents the prescribed turbulence intensity of 5% adopted for external aerodynamic applications, $U_\infty$ is the freestream inlet velocity of 48.69 m/s and *l* denotes the turbulence length scale which is specified as $0.07\,L_{\text {ref}}$ with $L_{\text {ref}}$ defined as the airfoil chord length^72,73^. A constant static pressure boundary condition ($p = 0$) was imposed at the outlet and the velocity and turbulence variables were subject to zero gradient conditions ($\partial u_i/\partial n = 0$, $\partial k/\partial n = 0$ and $\partial \omega /\partial n = 0$). A no-slip boundary condition and a zero normal pressure gradient ($\partial p / \partial n = 0$) were specified for all domain walls which includes the airfoil surface. For the turbulence variables, the SST $k{-}\omega$ model employs its standard near-wall treatment with the turbulent kinetic energy set to $k = 0$ and the specific dissipation rate defined as $\omega = 10\left( \frac{6\nu }{\beta _1 y^2}\right)$ where *y* is the wall-normal distance, $\nu$ denotes the kinematic viscosity and $\beta _1 = 0.075$^72,73^.

By providing consistent definitions of the flow and turbulence variables at every point within the computational domain the boundary conditions improve the numerical stability and increase the physical realism of the simulation results. Air was modeled as an incompressible fluid with a density of 1.184 kg/m$^3$ and a dynamic viscosity of $1.86 \times 10^{-5}$ Ns/m$^2$^74,75^. Pressure velocity coupling was achieved using the Semi-Implicit Method for Pressure Linked Equations (SIMPLE) algorithm. Momentum equations were discretized with a second order upwind scheme for improved accuracy and time advancement was performed using a first order implicit method. Additionally, a UDF were implemented to capture the impact of plasma actuation. Convergence was assessed by monitoring mass and momentum residuals with a convergence criterion set at $1.0 \times 10^{-7}$.

### Mesh sensitivity analysis

**Fig. 4:**
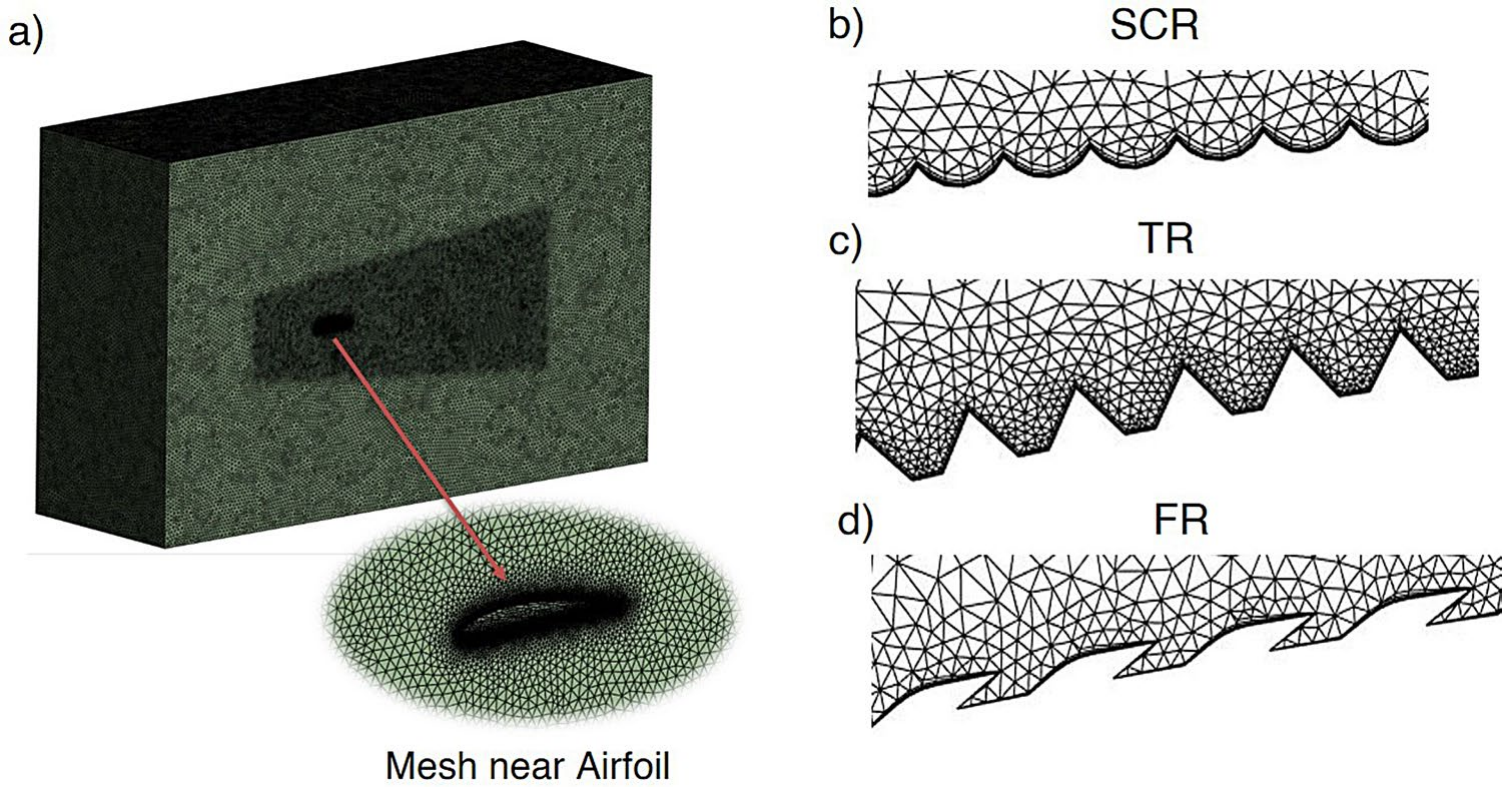
Computational domain with mesh and magnified view of airfoil with riblets.

Figure 4 illustrates the overall computational domain and the mesh strategy adopted for the mesh sensitivity analysis. Figure 4a shows the complete three-dimensional domain where a unstructured mesh is employed with progressive refinement towards the airfoil surface to ensure adequate near-wall resolution for accurately capturing the boundary layer development and riblet induced flow modifications. The airfoil is positioned centrally within the domain and the mesh density increases significantly in its vicinity to minimize numerical diffusion and to provide reliable predictions of separation and reattachment. Figure 4b–d present magnified views of the surface mesh corresponding to the three riblet geometries investigated in this study. These refinement near riblets highlight the fine discretization applied along the riblet contours which ensures sufficient grid clustering to resolve their small scale geometric features and the associated near-wall vortical structures.

**Fig. 5:**
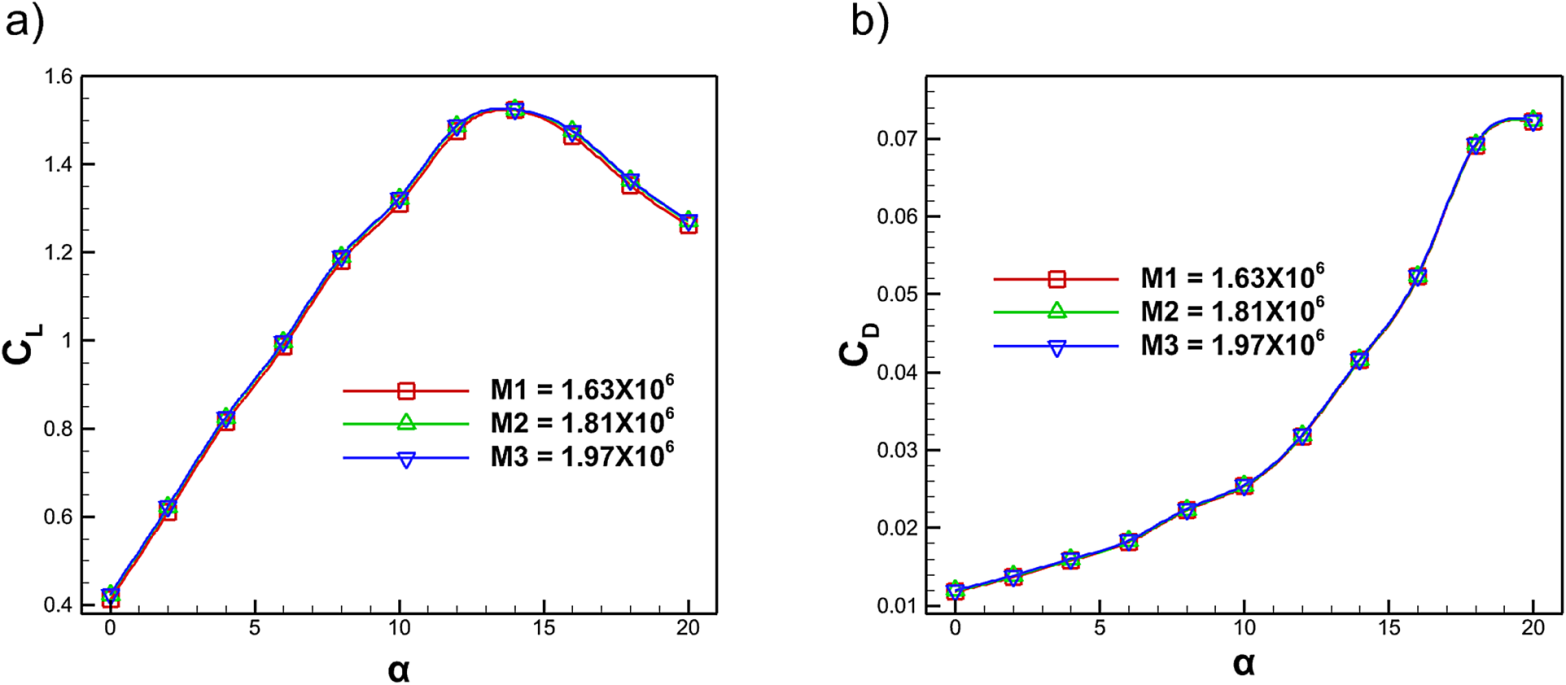
Mesh independence analysis of lift and drag coefficients of NACA4412 airfoil.

The accuracy and reliability of the numerical setup were ensured by performing a mesh independence study. Three meshes of coarse, medium and fine were generated using an unstructured grid . Local refinements were introduced through body sizing and edge sizing around the airfoil, particularly in the near-wall region, to adequately capture boundary layer behaviour and resolve critical flow features, as shown in Fig. 4. To effectively reduce drag and control flow separation, riblet heights must be sized to remain within the buffer layer where they inhibit spanwise motion of near wall vortical structures without extending into the logarithmic region^52–54^. Additionally, the near wall mesh was constructed using 20 inflation layers with a growth rate of 2 and the first-cell thicknesses for the coarse, medium and fine meshes were set to $1.08\times 10^{-5}\,\textrm{m}$, $7.1\times 10^{-6}\,\textrm{m}$, and $5.1\times 10^{-6}\,\textrm{m}$, respectively, to ensure appropriate $Y^{+}$ values for accurate turbulence modeling. The three mesh levels consisted of M1 with $1.63 \times 10^{6}$ elements, M2 with $1.81 \times 10^{6}$ elements and M3 with $1.97 \times 10^{6}$ elements. After convergence the maximum $Y^{+}$ values were found to be 1.24, 0.88 and 0.63 for M1, M2 and M3 respectively, confirming that the medium and fine meshes achieved sufficient viscous sublayer resolution. Figure 5 presents the difference in coefficient of lift and drag for the three mesh densities. These values showed negligible sensitivity to further mesh refinement so the maximum $C_{L}$ values were 1.523, 1.524 and 1.526 for M1, M2 and M3 while the corresponding $C_{D}$ values were 0.0415, 0.0416 and 0.0417. The observed $Y^{+}$ distribution further verified that both M2 and M3 provided the required near-wall accuracy for the SST *k*–$\omega$ turbulence model. Considering the marginal deviation in $C_{L}$ and $C_{D}$ together with the computational efficiency the mesh (M2), containing $1.81 \times 10^{6}$ elements was chosen for all remaining simulations as it maintains $Y^{+} < 1$ and produces aerodynamic results that are independent of further mesh refinement.

Mesh independence study was carried out for the fillet riblet configuration at an angle of attack of $14^\circ$ using three successively refined grids comprising M1 with $1.63 \times 10^{6}$ elements, M2 with $1.81 \times 10^{6}$ elements and M3 with $1.97 \times 10^{6}$ elements. The corresponding lift and drag coefficients are listed in Table 1. As the mesh is refined, the lift coefficient increases from 1.612 for M1, 1.639 for M2 finally 1.640 for M3 with the variation between the medium and fine meshes being less than 0.07% which indicates that the lift prediction has effectively converged. A similar behaviour is observed for the drag coefficient which stabilizes between the M2 and M3 grids with a change of less than 0.5%. These small variations confirm that further mesh refinement does not lead to any appreciable improvement in accuracy. Hence, the mesh containing $1.81 \times 10^{6}$ elements is selected for the final simulations, offering an optimal trade-off between solution accuracy and computational cost.

**Table 1:** Mesh independence study for the fillet riblet configuration at $14^\circ$ AOA.

Mesh Elements	C$_L$	C$_D$
1.63 $\times 10^{6}$	1.612	0.0389
1.81 $\times 10^{6}$	1.639	0.0408
1.97 $\times 10^{6}$	1.640	0.0410

The temporal accuracy and numerical stability of the unsteady flow simulations were evaluated using a time-step sensitivity investigation at the high lift condition of $\alpha = 16^\circ$. The lift and drag coefficients for the three distinct time step values $2.5 \times 10^{-2}$, $2.5 \times 10^{-3}$ and $2.5 \times 10^{-4}$ that were shown in Table 2. The smallest time step, $\Delta t = 2.5 \times 10^{-4}$, was treated as the reference solution for evaluating the temporal discretization error. Additionally, the total physical simulation time was set to 16 s to ensure statistically converged aerodynamic loads and the time-averaged coefficients were computed over a average sampling window of 5–16 s^76.^

**Table 2:** Time-step sensitivity of the predicted lift coefficient for the NACA4412 airfoil.

Time step size ($\Delta t$)	$C_L$	Error in (%)
$2.5 \times 10^{-4}$	1.481	–
$2.5 \times 10^{-3}$	1.477	0.27
$2.5 \times 10^{-2}$	1.316	10.90

As presented in Table 2 the changes in $C_L$ diminish systematically with successive time-step refinement which confirming temporal convergence of the solution. Decreasing the time step from $2.5 \times 10^{-2}$ to $2.5 \times 10^{-3}$ led to an increase in the lift coefficient from 1.316 to 1.481 which accompanied by a significant reduction in error from 10.90% to 0.27%. The difference between the solutions obtained with $\Delta t = 2.5\times 10^{-3}$ and $\Delta t = 2.5\times 10^{-4}$ was less than 0.3% and demonstrating that additional time-step refinement would yield negligible improvement while significantly increasing computational expense. Hence a time step of $2.5\times 10^{-3}$ was adopted for all remaining simulations which offering an effective compromise between numerical accuracy and computational efficiency.

### Validation

**Fig. 6:**
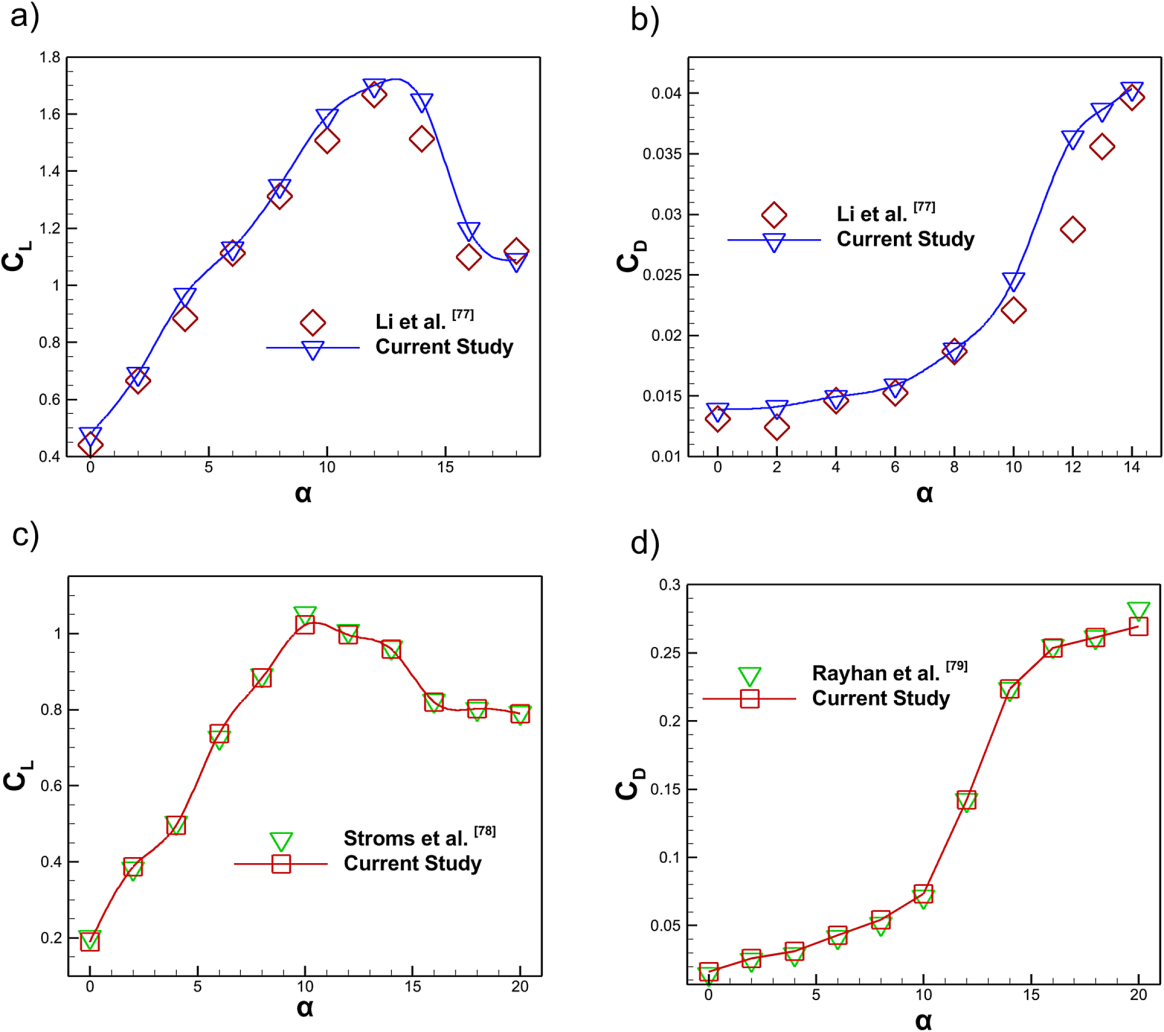
Comparison of computed aerodynamics coefficients with experimental data.

To ensure the reliability of the numerical approach the present model was validated against benchmark experimental data^77^ for a NACA0012 airfoil with a chord length of 1 m at a Reynolds number of $2.1\times 10^6$ over a wide range of angles of attack 0$^{\circ }$ - 18$^{\circ }$. The comparison of the $C_L$ shown in Fig. 6a indicates very good agreement between the numerical and experimental results^77^. The predicted maximum lift at 14$^{\circ }$ was only  1.4 % higher than the experimental value which demonstrates the capability of the turbulence model to accurately resolve pre-stall flow physics and aerodynamic load development. Furthermore, the slope of the computed $C_L$ curve was found to be consistent with that of the experiments, confirming the ability of the model to capture the incremental growth of lift with angle of attack. The $C_D$ comparison with experimental results shown in Fig. 6b revealed a minor under prediction at 10$^{\circ }$, primarily attributed to reduced flow separation in the numerical solution. Beyond 12$^{\circ }$, though, the calculated $C_D$ closely tracked the experiment trend^77^, suggesting that the model was capable of simulating the post stall aerodynamic behavior with good accuracy. Quantitatively, the mean deviation between computation and experiment was  1.4% for $C_L$ and  1.8% for $C_D$ indicating its capability in illustrating the aerodynamic performance of the airfoil tested in this research.

Furthermore, a validation analysis was carried out by comparing the baseline NACA4412 airfoil computed lift and drag coefficients with experimental data found in the literature^78,79^. Figure 6c presents the variation of the predicted $C_L$ alongside the measurements of Storms et al.^78^ at a Reynolds number of $3.8\times 10^6$, while Fig. 6d compares the computed $C_D$ values with the experimental results of Rayhan et al. ^79^ at a Reynolds number of $4.2\times 10^5$. The predicted aerodynamic coefficients compare well with both reference datasets across all AOA. The maximum $C_L$ obtained in the present simulation is $C_{L} = 1.031$ at $\alpha = 10^\circ$ which is in close accordance with the experimental value of $C_{L} = 1.049$ published by Storms et al.^78^. The computed aerodynamic coefficients were compared with wide range of angles of attack up to $20^\circ$ covering both pre-stall and post-stall regimes. The numerical predictions demonstrate good agreement with the experimental lift and drag trends. The general trends in both lift and drag coefficients are well captured which demonstrates that the chosen computational approach and mesh resolution can reliably predict the aerodynamic response of the NACA4412 airfoil.

**Fig. 7:**
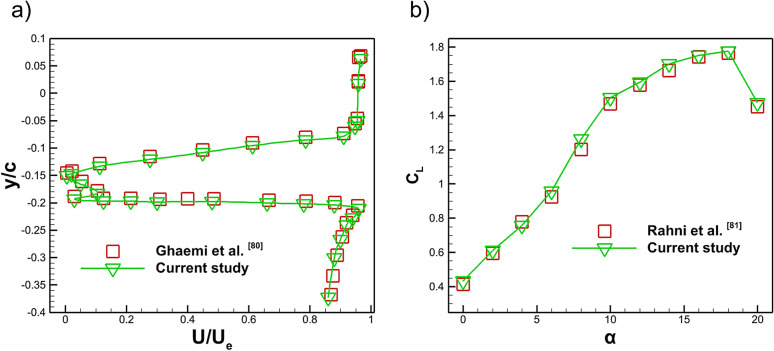
**a** Comparison of wake velocity profile at 14$^\circ$, **b** Comparison of computed and experimental lift coefficient of plasma actuator.

The wake velocity profile at an angle of attack of $14^\circ$ is further assessed by comparing the dimensionless streamwise velocity profile ($U/U_{e}$) at the trailing edge of the NACA 4412 airfoil with the experimental data of Ghaemi et al.^80^ at a Reynolds number of $4\times 10^{5}$, as illustrated in Fig. 7a. The experimental data and present simulation data of trailing edge wake generation plotted against the normalized vertical coordinate *y*/*c*. Despite the flow complexity associated with a high AOA condition where strong adverse pressure gradients, boundary layer thickening and partial separation typically distort the wake structure. The present CFD results successfully reproduce the key wake characteristics reported in the reference study. Specifically, the simulation accurately tracks the reduction in velocity deficit in the upper part of the wake were positive *y*/*c*, captures the nearly symmetric deficit distribution around the mid-wake region and matches the sharper drop in normalized velocity in the lower portion of the wake. The close agreement across the entire wake thickness confirms that the present numerical model captures both the magnitude and shape of the velocity deficit with high fidelity. Figure 7b presents the validation of the lift coefficient for a NACA4412 airfoil equipped with a plasma actuator over a range of angles of attack from 0$^\circ$ to 20$^\circ$ at a Reynolds number of $1 \times 10^{6}$. The experimental measurements of Rahni et al.^81^ are compared with the present numerical results. The maximum $C_L$ attained in the present simulation is $C_{L} = 1.776$ at $\alpha = 18^\circ$, which is in close accordance with the experimental value of $C_{L} = 1.763$ reported by Rahni et al.^81^. A strong agreement is observed across the entire AOA range, particularly in the linear lift region and near stall where the CFD accurately captures both the peak lift and its subsequent reduction. This close correspondence demonstrates that the plasma actuator modeling and stall behavior are well reproduced by the simulation. Overall, the comparison confirms that the present numerical approach reliably predicts the lift enhancement characteristics of the plasma actuated NACA4412 airfoil.

## Results and discussion

This section discusses the results obtained from the investigation of hybrid flow control techniques applied to the NACA4412 airfoil. Bio-inspired riblet structures were positioned on the upper surface of the airfoil to passively manipulate near wall turbulence while AC-DBD plasma actuators were implemented near the leading edge which actively control separation of flow and energize the boundary layer. Analyses of vorticity magnitude, streamline distribution and aerodynamic force coefficients revealed that the hybrid approach improved $C_L$ generation, reduced $C_D$ and enhanced the $C_L$/$C_D$ ratio, particularly at higher angles of attack. The riblets contributed to stabilizing the vortical structures and minimizing wake thickness while plasma actuation supported flow attachment. In addition, ANN based SCG and SVM models predicted aerodynamic coefficients very accurately with a close match to CFD data, highlighting their potential for quick performance assessment.

### Impact of riblets with AC-DBD

**Fig. 8:**
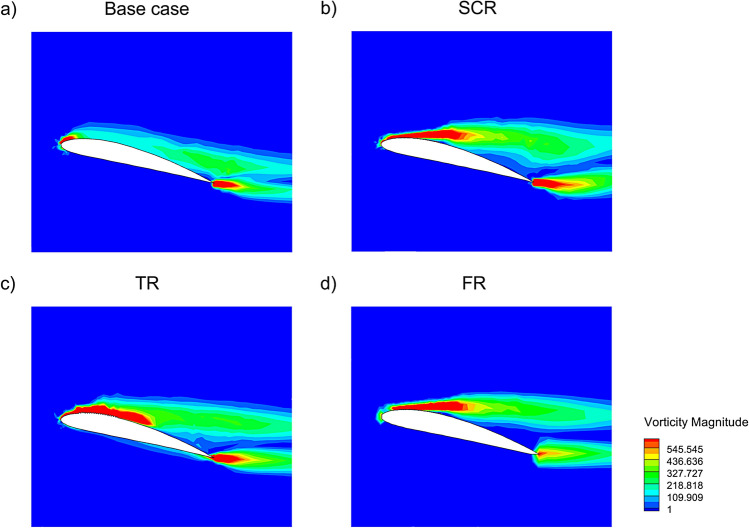
Vorticity magnitude of NACA4412 airfoil with riblet at 14$^{\circ }$ AOA.

Figure 8 shows the contours of vorticity magnitude of NACA4412 airfoil at 14$^{\circ }$ AOA demonstrating the effect of different riblet configurations. In the base case, Fig. 8a the flow separates early giving rise to a wider wake and weaker near wall vorticity which leads to higher total $C_D$ primarily due to elevated pressure and skin friction components. The SCR configuration in Fig. 8b introduces rounded riblet grooves interacting with the boundary layer to suppress turbulent bursts and reduce the near wall shear which leads to a noticeable reduction in skin friction drag. The TR configuration in Fig. 8c with sharp riblet tips enhances streamwise vortex formation that disrupts transverse turbulent transport. This suppresses wall normal velocity fluctuations and leads to the maximum reduction in skin friction drag although it may increase local turbulence intensity. In the FR case in Fig. 8d, the bio inspired fillet shaped riblets gently guide the flow which stabilizes the near wall region and achieving a balanced reduction in skin friction drag with improved flow attachment and a narrower wake. Overall the incorporation of riblets on the upper surface of the airfoil is an effective passive technique to reduce skin friction drag by modifying near wall turbulence structures.

**Fig. 9:**
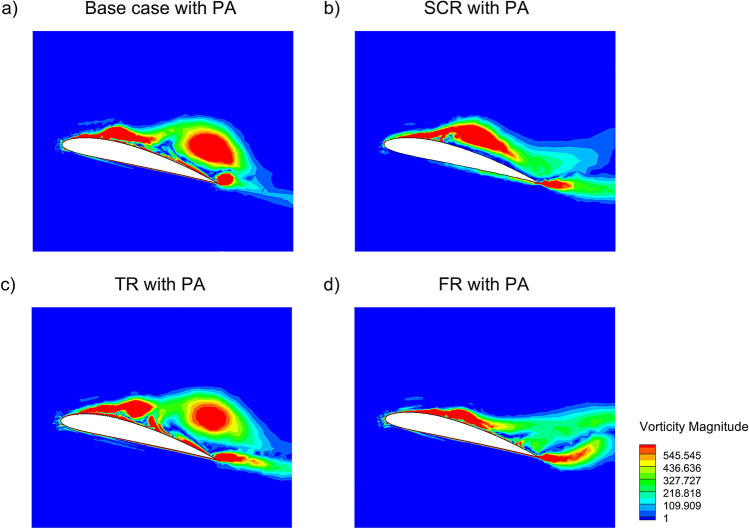
Vorticity magnitude of NACA4412 airfoil with riblet and PA at 14$^{\circ }$ AOA.

The vorticity magnitude contours in Fig. 9 illustrate the effect of integrating a plasma actuator with riblet configuration at 14$^{\circ }$ angle of attack. In the base case with PA in Fig. 9a, the plasma actuation energizes the boundary layer which partially suppressing flow separation and narrowing the wake, although skin friction remains elevated due to the lack of riblet control. The SCR with PA case in Fig. 9b exhibits better vorticity confinement near the surface and a thinner turbulent shear layer which indicates a synergistic interaction between the riblet induced streamwise alignment and the actuator flow energization where results in reduced skin friction drag compared to the base case. The TR with PA configuration in Fig. 9c shows intense and well structured near wall vorticity patterns which confirms enhanced shear suppression and more pronounced drag reduction, owing to the strong alignment of actuator induced momentum and riblet generated vortices. The FR with PA case in Fig. 9d offers well distributed and stable vorticity field with reduced separation and improved wake symmetry. This behavior helps preserve boundary layer integrity along the chord and results in a moderate reduction in skin friction drag. Overall, the combination of riblets with plasma actuation significantly enhances skin friction drag reduction by manipulating near wall turbulence and delaying flow separation more effectively than passive or active control alone.

**Fig. 10:**
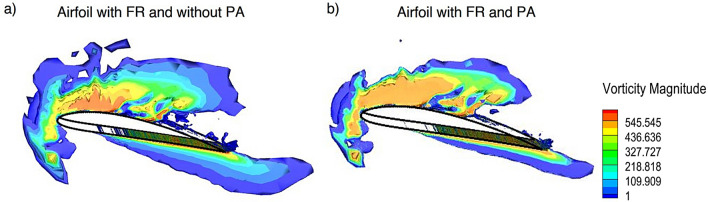
Vorticity magnitude of NACA4412 airfoil with, **a** Fillet riblet and **b** Fillet riblet with PA.

Figure 10 depicts the 3D vorticity magnitude distribution for the NACA4412 airfoil under two configurations at 16$^{\circ }$ AOA. Figure 10a with fillet riblets only and Fig. 10b with both FR and plasma actuator. In normal airfoil the flow field exhibits pronounced large scale vortical structures and early flow separation near the suction surface which results in a thicker wake region and elevated vorticity levels downstream. This is indicative of unsteady and turbulent flow conditions which contribute to increased pressure drag. While FR shown in Fig. 10a as a passive control mechanism provides some enhancement by disrupting spanwise vortex formation and channeling high energy fluid close to the surface and its ability to fully suppress separation is limited under adverse pressure gradients. In contrast, Fig. 10b demonstrates a significantly altered flow structure with more attached streamlines, reduced wake turbulence and a notable suppression of vortex shedding due to the activation of PA. Acting as an active flow-control mechanism the PA introduces body forces that energize the boundary layer thereby promoting flow attachment and reducing the extent of separation. The combination of FR with PA leads to a measurable increase in the $C_L$ with a concurrent reduction in the $C_D$. The boundary layer suppressed by the FR sustains higher suction on the upper surface of the airfoil which increasing lift while the reduction in wake thickness and turbulence directly lowers both pressure and skin friction drag. The combined implementation of bio-inspired FR and PA was thus found to significantly enhance the aerodynamic performance through improvement in boundary layer stability, minimizing flow separation and optimizing the $C_L$/$C_D$ ratio of the NACA4412 airfoil.

**Fig. 11:**
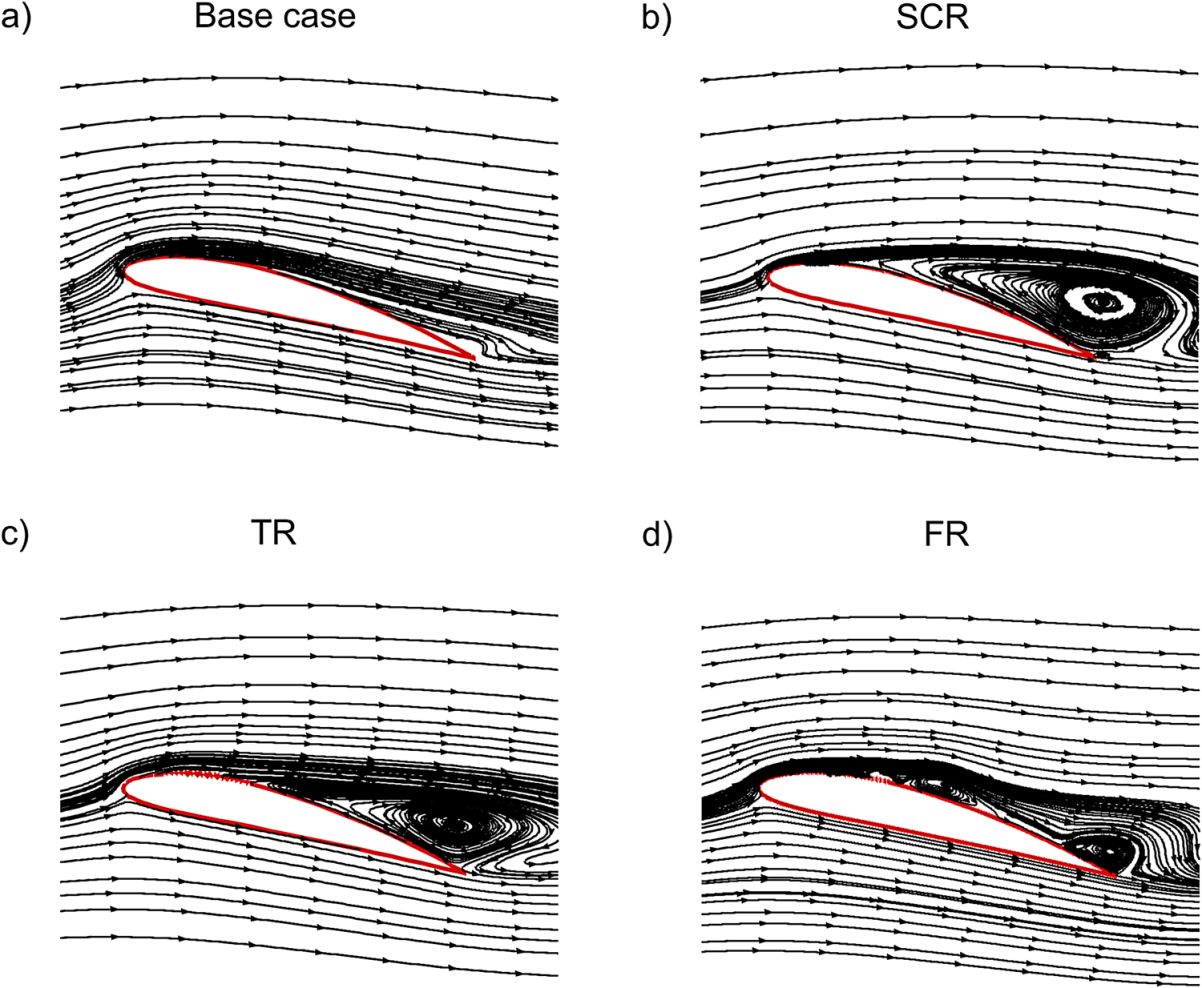
Streamlines of the NACA4412 airfoil equipped with a riblet at 14$^{\circ }$ AOA.

At an angle of attack of 14$^{\circ }$ the streamline patterns in Fig. 11 highlight the influence of different riblet geometries on the NACA4412 airfoil flow characteristics. In the base case, Fig. 11a shows a separation region develops near the trailing edge due to the strong adverse pressure gradient which produces a moderately thick wake. With the semi-circular riblet in Fig. 11b, the circular geometry induces an early separation and a large recirculation bubble downstream of the riblet which pushes the outer streamlines away from the surface and resulting in a thicker wake which is aerodynamically unfavorable. In contrast, the triangular riblet in Fig. 11c sharpens the flow response by generating a compact recirculation bubble with rapid reattachment which entrains high momentum fluid toward the wall, delays trailing edge separation and reduces wake thickness relative to the base case. The fillet riblet in Fig. 11d further improves flow behavior by softening the geometric gradients while retaining vortex energization results in the shortest recirculation region, the most attached suction side flow and the thinnest wake among all cases. Overall, the separation bubble size and wake thickness are largest for the semi-circular riblet followed by the base case then the triangular riblet and finally the fillet riblet. Among these, the bio inspired fillet riblet provides the most favorable aerodynamic performance by maintaining better flow attachment, minimizing wake thickness and enhancing $C_L$/$C_D$ characteristics at this angle of attack.

**Fig. 12:**
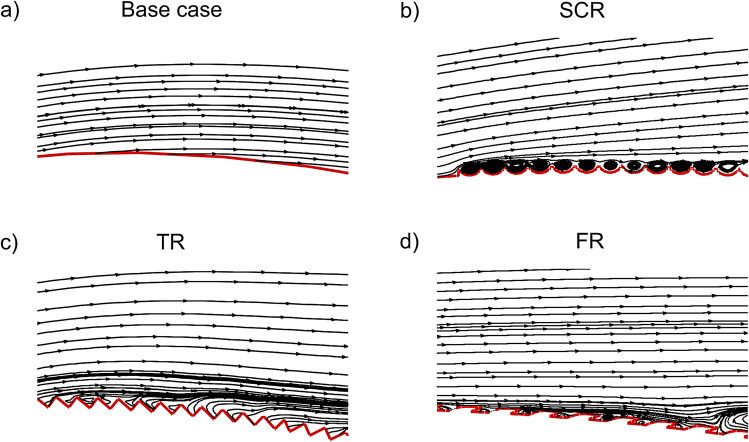
Streamlines of upper surface of the NACA4412 airfoil equipped with riblets.

The streamline plots in Fig. 12 provide a magnified view of the near wall flow structures formed over the upper surface of the NACA4412 airfoil at 14$^{\circ }$ angle of attack with various riblets. In the base case Fig. 12a, the streamlines are relatively parallel and near the surface, indicating a fully developed boundary layer with no mechanisms present to suppress wall shear stress or inhibit turbulent burst events, resulting in higher skin friction drag. The SCR configuration in Fig. 12b shows small recirculating zones within the grooves where the riblet cavities trap low momentum fluid. This helps isolate the wall from turbulent cross flow which leads to moderate drag reduction by decreasing wall normal momentum transport. In the TR case shown in Fig. 12c, sharp riblet peaks create strong secondary vortices and significant interaction with the turbulent boundary layer. These spanwise vortical structures restrict transverse motion of eddies and channel flow along the riblet direction by producing the most effective passive reduction in skin friction drag. The FR configuration in Fig. 12d exhibits smoother longitudinal streaks and subtle recirculations near the wall. The bio inspired fillet riblet guides the flow gently while still creating a textured boundary that stabilizes the near wall region and offering balanced drag reduction with relatively low turbulence amplification.

**Fig. 13:**
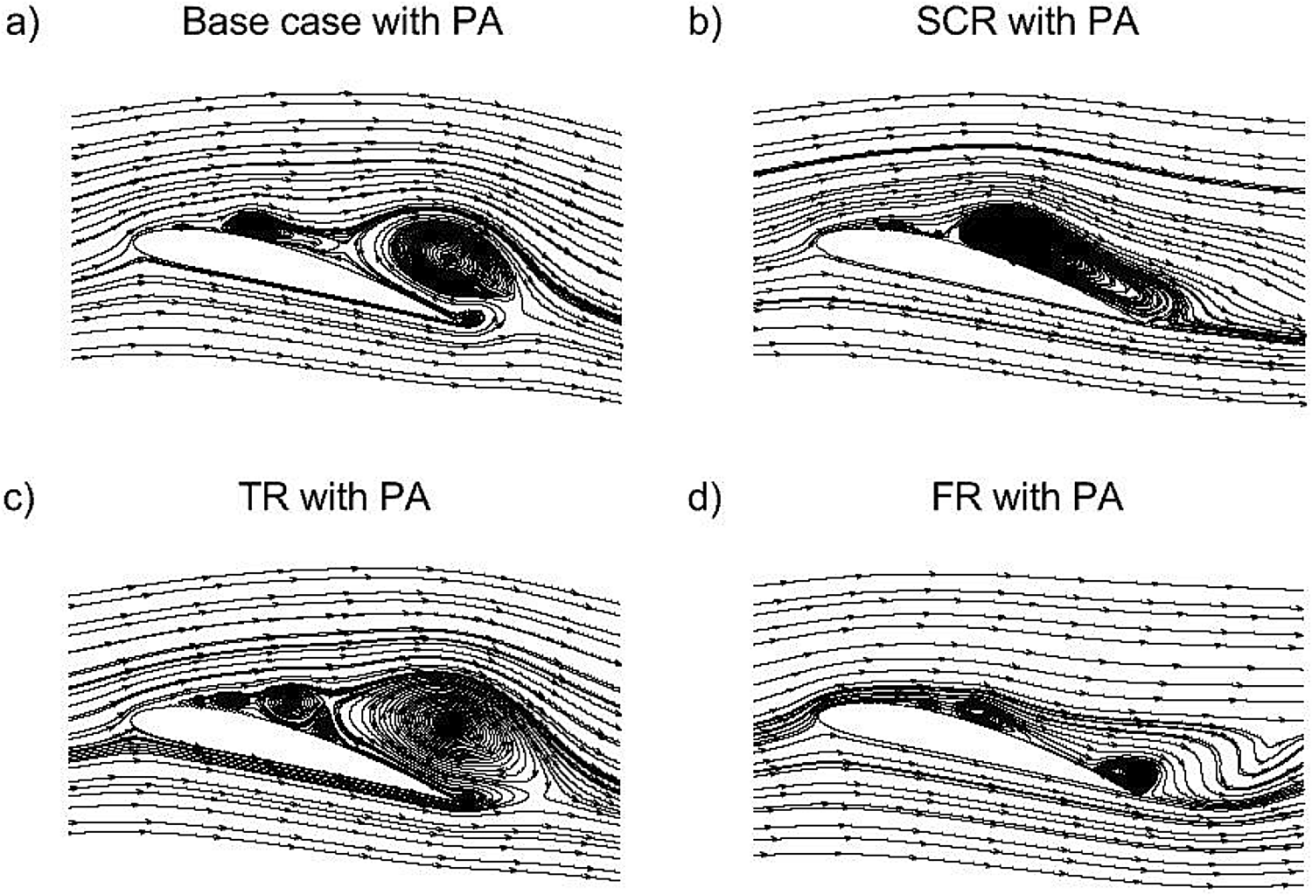
Streamlines of the NACA4412 airfoil equipped with riblet and plasma actuator at 14$^{\circ }$ AOA.

Figure 13 presents the streamline fields for the different riblet with plasma actuator configurations at 14$^{\circ }$. Figure 13a illustrates the base case with PA in which the injected momentum reduces the size of the separation bubble near leading edge and serves as a reference for assessing the combined effect of riblets. Figure13b shows the semi-circular riblets with PA where the smooth curvature of the riblets helps guide the near-wall flow more effectively and leads to a noticeable reduction in recirculation compared with the base case. In Fig. 13c, the triangular riblets with PA generate sharper flow deviations that intensify small-scale vortical interactions which resulting in a slightly larger and more turbulent separation bubble relative to the SCR configuration. Figure 13d presents the fillet riblets with PA whose blended sharp and smooth geometric features promote improved streamline attachment and reduced reverse flow when compared with the TR case. When compared against their corresponding cases without PA, all PA assisted configurations demonstrate significantly smaller separation regions, smoother streamline curvature near leading edge and enhanced reattachment behavior. The findings emphasize the synergistic interaction between the plasma actuator and the riblets where the actuator strengthens the boundary layer while the riblets optimize wall flow organization and collectively offering superior aerodynamic control.

**Fig. 14:**
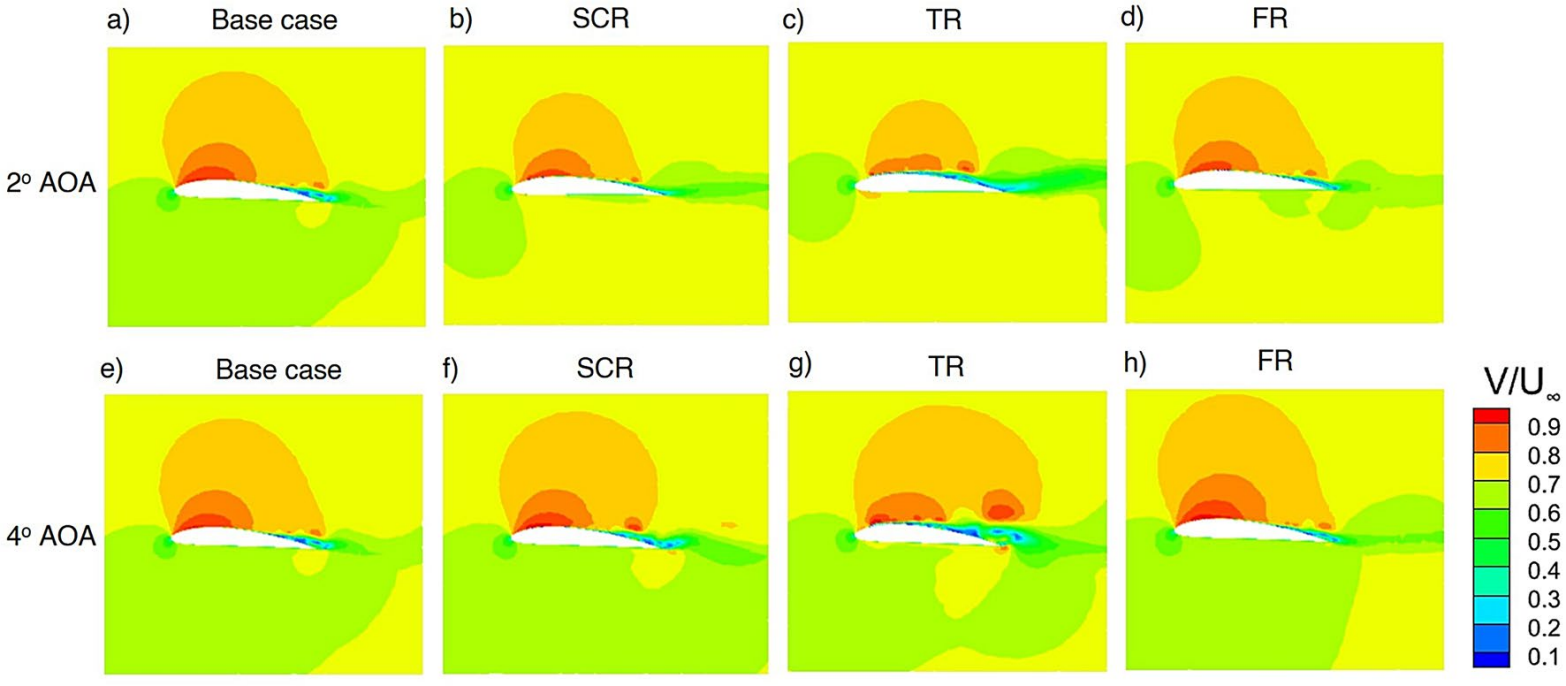
Velocity magnitude contour of airfoil with different riblets cases at 2$^\circ$ and 4$^\circ$ AOA.

Figure 14 shows velocity contours ($V/U_{\infty }$) around a NACA 4412 airfoil fitted with three riblet configurations at 2$^\circ$ AOA and 4 $^\circ$ AOA illustrating the effect of increasing incidence on flow behaviour and riblet performance. At 2$^\circ$ AOA Fig.14a-d, the baseline airfoil exhibits a small region of mild velocity deficit above the suction surface which indicates a very weak shear layer but no significant flow separation at this low incidence. The wake remains thin and stable which shows that the flow is fully attached. The semi-circular riblet enhances near wall organization of the boundary layer and slightly reduces the vertical velocity gradients near the 25% chord location. A minor reduction in the upper surface shear layer thickness is visible which indicates improved momentum exchange and delayed onset of separation. The triangular riblet introduces stronger shear layer energization downstream of its location and producing a more pronounced acceleration region compared to SCR. Therefore this configuration increases the boundary layer resistance to adverse pressure gradients even at small angles of attack and hence further improves near wall mixing. The fillet riblet provides the smoothest boundary layer transition due to reduced sharp shear gradients while maintaining a more uniform near wall velocity distribution. As a result the wake becomes thinner and indicates improved aerodynamic stability with reduced surface shear disturbances.

At 4$^\circ$ AOA Fig. 14e–h, the baseline airfoil shows stronger adverse pressure gradients appear near mid chord by creating a more visible separated shear layer above the suction surface. The wake expands are noticeably compared to 2$^\circ$ AOA which indicates a partial separation bubble and reduced aerodynamic efficiency. The semi-circular riblet weakens the separation zone by introducing momentum into the near wall region. The separated bubble becomes smaller than in the baseline case and the wake decreases in height by demonstrating delayed separation and better flow stability compared to the smooth surface. The triangular riblet generates the strongest energization of the boundary layer through its sharper rib geometry. This results in a significantly reduced separation region and a more contracted wake. The pronounced mixing assists the airfoil in maintaining attached flow at this higher AOA. The fillet riblet again produces the most uniform control effect. It smoothens the velocity contours over the suction surface which substantially limiting the separated region and producing the smallest wake among all cases. Its gradual shape continuously supplies momentum to the boundary layer by maintaining strong attachment even under increased aerodynamic loading.

**Fig. 15:**
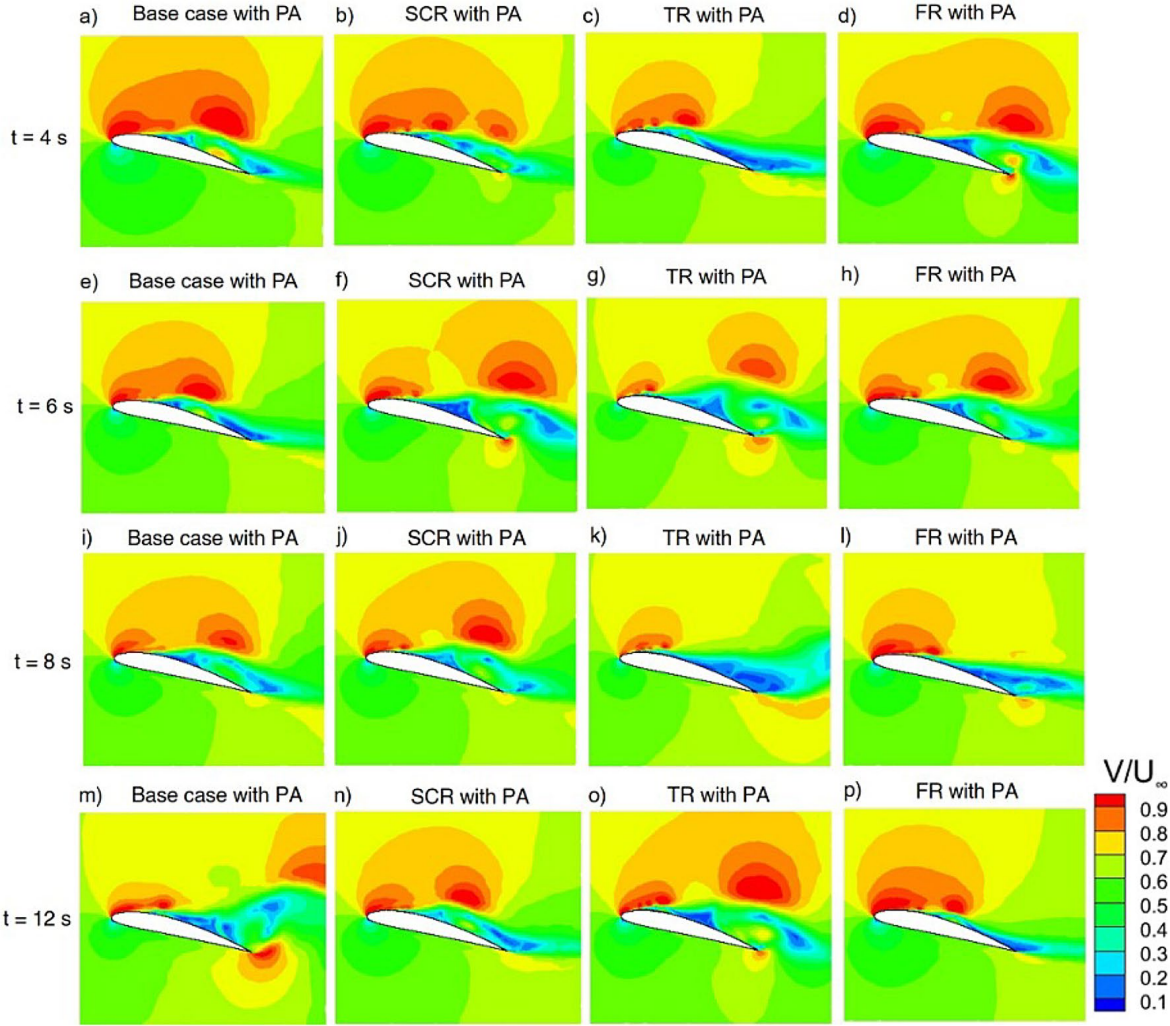
Velocity magnitude contour of NACA4412 airfoil with riblets and plasma actuator at 14 $^\circ$ AOA.

Figure 15 presents the time resolved evolution of the velocity contours around the airfoil for the baseline with riblet and plasma actuator. The contours are shown at $t = 4~\textrm{s}$, $6~\textrm{s}$, $8~\textrm{s}$ and $12~\textrm{s}$ to examine the transient response and long term effectiveness of plasma-assisted flow control. At $t = 4~\textrm{s}$, plasma actuation generates a localized high-momentum region near the leading edge on the suction surface which indicates effective boundary layer energization in all cases. However the baseline configuration still exhibits a relatively thick low velocity region downstream and suggesting incomplete suppression of separation. At $t = 6~\textrm{s}$ and $8~\textrm{s}$ distinct differences show up between the configurations. While the baseline and SCR cases still have apparent wake deficits the TR and FR configurations exhibit increased momentum change along the suction surface which leads to thinner shear layers and reduced wake widths. Among all cases the FR configuration consistently demonstrates the smallest low velocity region and the smoothest velocity recovery which indicates improved boundary layer stabilization and delayed separation. By $t = 12~\textrm{s}$ plasma actuation with riblet assisted cases particularly the FR configuration maintain a compact wake and uniform velocity distribution. The SCR case provides moderate stabilization and the TR case yields stronger wake contraction and momentum retention. Overall, the temporal evolution of the velocity contours demonstrates that plasma actuation primarily enhances th near wall flow near the leading edge region while riblets govern the downstream persistence and stability of this energization. The combined use of plasma actuation and riblets especially with the fillet riblet configuration delivering the most reliable and time consistent flow control performance as evidenced by delayed separation and reduced wake deficit.

**Fig. 16:**
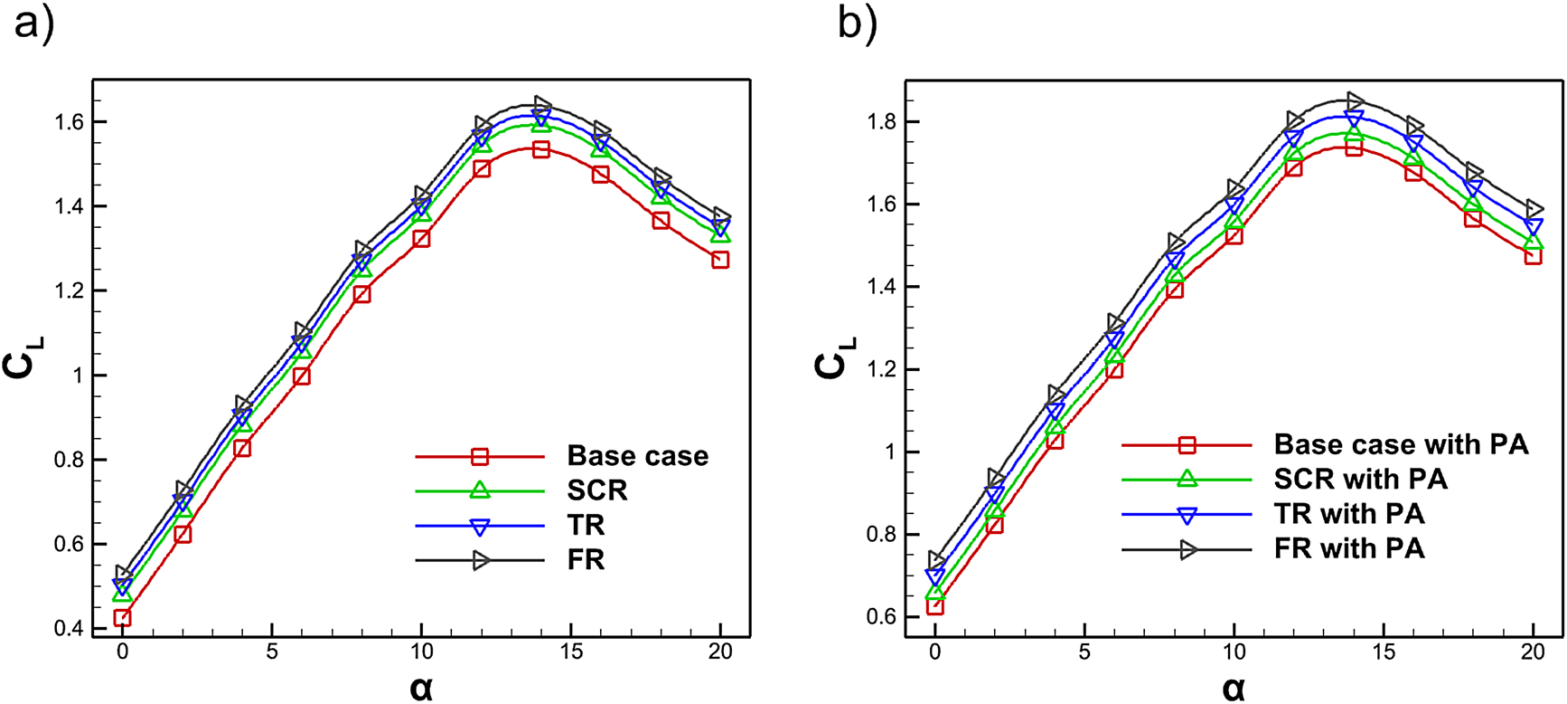
Variation of $C_L$ with AOA for the base case and riblet equipped airfoil **a** without PA **b** with PA.

Figure 16 illustrates the variation in the lift coefficient of the NACA4412 airfoil under different riblet configurations and highlighting the influence of the plasma actuator on aerodynamic performance. Figure 16a compares the $C_L$ for the base case with the semi-circular, triangular and fillet riblet configurations. The results indicate that the maximum $C_L$ increases by 3.63%, 5.09% and 6.71% for the semi-circular, triangular and fillet riblets respectively when compared to the baseline. The results demonstrate that riblet surface texturing enhances aerodynamic performance with the fillet riblet providing the greatest lift improvement due to its streamlined curvature. The triangular riblet also contributes significantly by promoting near wall momentum exchange while the semi-circular riblet offers a moderate benefit. Overall, riblet geometries effectively improve lift by passively modifying boundary layer behavior. Figure 16b compares the $C_L$ for the base case, the base case with plasma actuation and hybrid configurations incorporating PA with semi-circular, triangular and fillet riblets. The findings indicate that the maximum $C_L$ increases by 13.01% with PA alone and further increases by 15.22%, 17.91% and 20.38% when combined with semi-circular, triangular and fillet riblets, respectively. The predicted $C_L$ values show excellent agreement with the benchmark data and previous investigations^82,83^. These results demonstrate the effectiveness of hybrid flow control where integrating riblet geometries with plasma actuation yields synergistic aerodynamic benefits. The bio inspired fillet riblet combined with plasma actuation provides the greatest lift enhancement by improving boundary layer control and delaying separation while the triangular and semi-circular configurations also contribute notably through enhanced near wall flow dynamics.

**Fig. 17:**
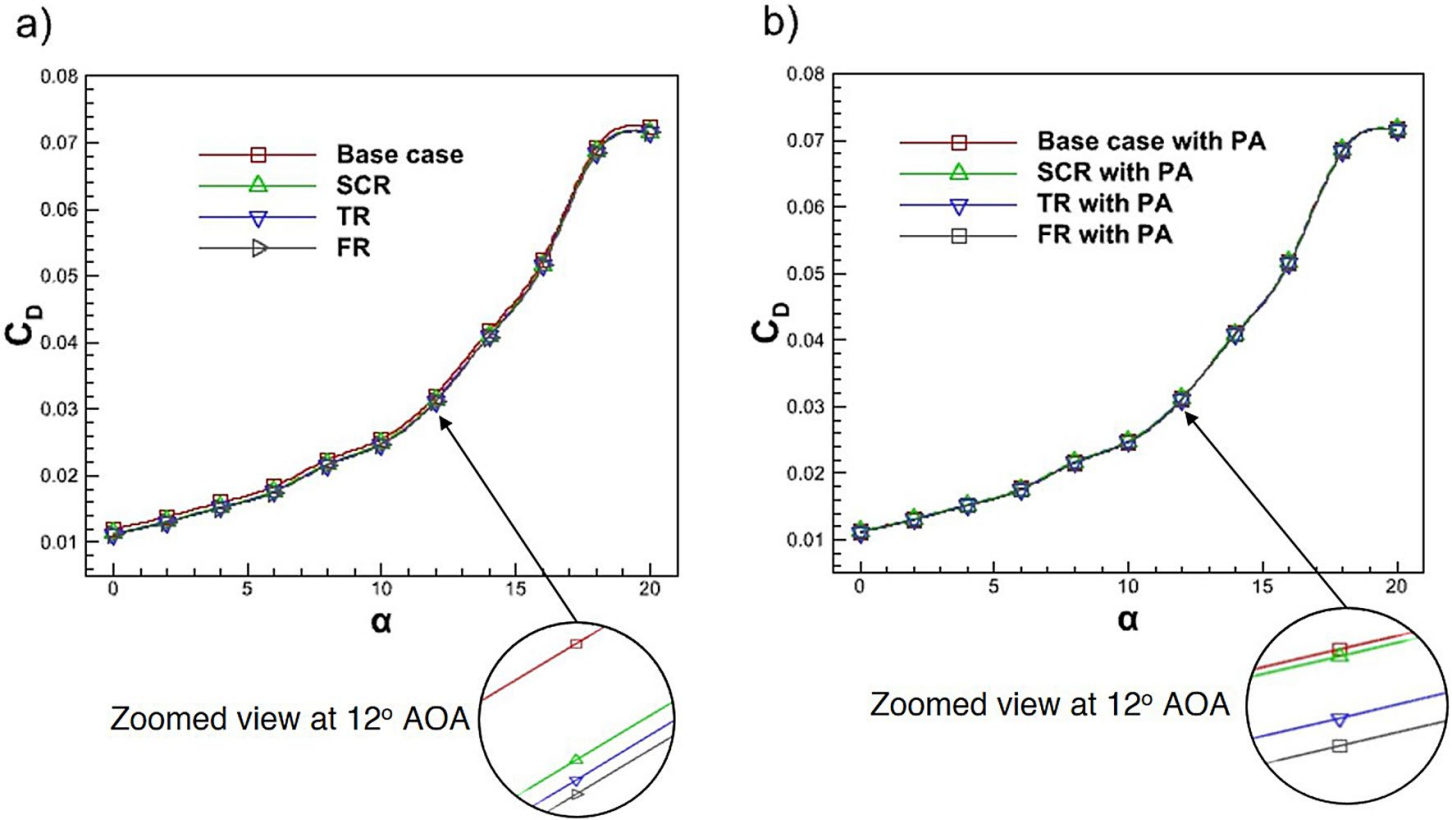
Variation of $C_D$ with AOA for the base case and riblet equipped airfoil **a** without PA **b** with PA.

Figure 17a presents the comparison of $C_D$ for the base case and various riblet configurations. The base case exhibits the highest $C_D$ while the implementation of riblet geometries semi-circular, triangular and fillet leads to progressive reductions in $C_D$. Specifically, the $C_D$ is reduced by 4.70%, 5.50% and 6.08% for the semi-circular, triangular and fillet riblet configurations, respectively. These reductions indicate the effectiveness of riblet structures in suppressing near wall turbulence and reducing skin friction drag. Figure 17b illustrates the $C_D$ comparison for the base case with plasma actuation and the hybrid configurations combining PA with semi-circular, triangular and fillet riblets. The application of PA alone results in a $C_D$ reduction of 4.78% relative to the baseline. Further reductions are observed when PA is integrated with riblet geometries are 4.87%, 5.79% and 6.20% for the SCR with PA, TR with PA and FR with PA configurations respectively. The predicted $C_D$ values show excellent agreement with the benchmark data and previous investigations^82,83^. The results show that the combination of riblet and PA flow control strategies reduce $C_D$. The zoomed view at 12$^{\circ }$ AOA is displayed in Fig. 17. The zoom image emphasizes the small known variations in the drag coefficient $C_D$ for all configurations. At this angle of attack the baseline configuration has the highest $C_D$ while all configurations with riblets exhibit a small amount of drag reduction. The fillet riblet provides the lowest $C_D$. While the absolute $C_D$ differences are small so they all have a noticeable and consistent trend shown in the inset. Each configuration achieves a uniform reduction in $C_D$ through plasma actuation therefore the configuration with the bio-inspired fillet riblet with plasma actuation to achieve the highest reduction in $C_D$. The significant reductions in skin friction drag from the fillet riblet configuration demonstrates the ability of the riblet with PA configuration to sustain a more uniform flow and provide the lowest skin friction drag. Thus, the results indicate that the hybrid combination will be more aerodynamically efficient for flow control.

**Fig. 18:**
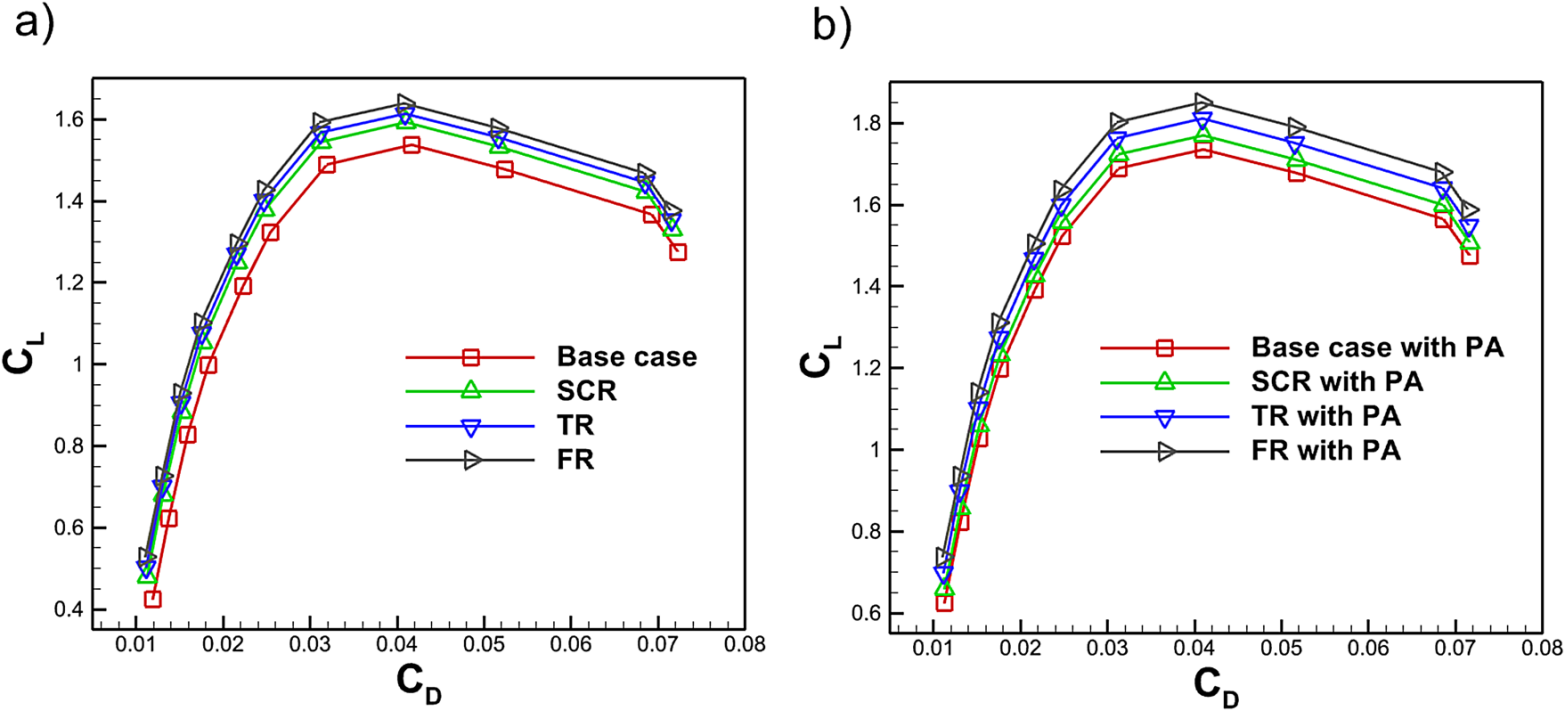
Variation of $C_D$ with $C_L$ for the base case and riblet equipped airfoil **a** without PA **b** with PA.

Figure 18 presents the $C_D$ with $C_L$ characteristics of the NACA4412 airfoil with various riblet geometries both without and with a plasma actuator present. Without an actuator in Fig. 18a the baseline case achieves a $C_L$ of 1.53 with a $C_D$ of 0.0416. The addition of riblets improves performance for semi circular, triangular and fillet geometries by increasing maximum $C_L$ to 1.59, 1.61 and 1.63 respectively with simultaneous $C_D$ reduction to 0.0410, 0.0409 and 0.0408. This confirms that riblet geometry plays a role in the reduction of $C_D$ as well as providing a modest increase in lift. When a plasma actuator is applied in combination with riblets shown in Fig. 18b further aerodynamic gains are observed. The baseline with an actuator reaches a maximum $C_L$ of 1.73 and $C_D$ of 0.043 while the semi-circular and triangular riblets yield $C_L$ values of 1.76 and 1.81 with corresponding $C_D$ values of 0.0409 and 0.0408. The best performance is achieved by the bio inspired fillet riblet with a plasma actuator which delivers the highest lift of 1.84 and the lowest $C_D$ of 0.0408. The computed numerical values show excellent agreement with the benchmark data and previous investigations^82,83^. These results demonstrate that riblets reduces $C_D$ and slightly enhance lift while the integration of a plasma actuator amplifies these benefits. The fillet riblet with actuator configuration providing the most favorable aerodynamic characteristics at higher angles of attack.

**Fig. 19:**
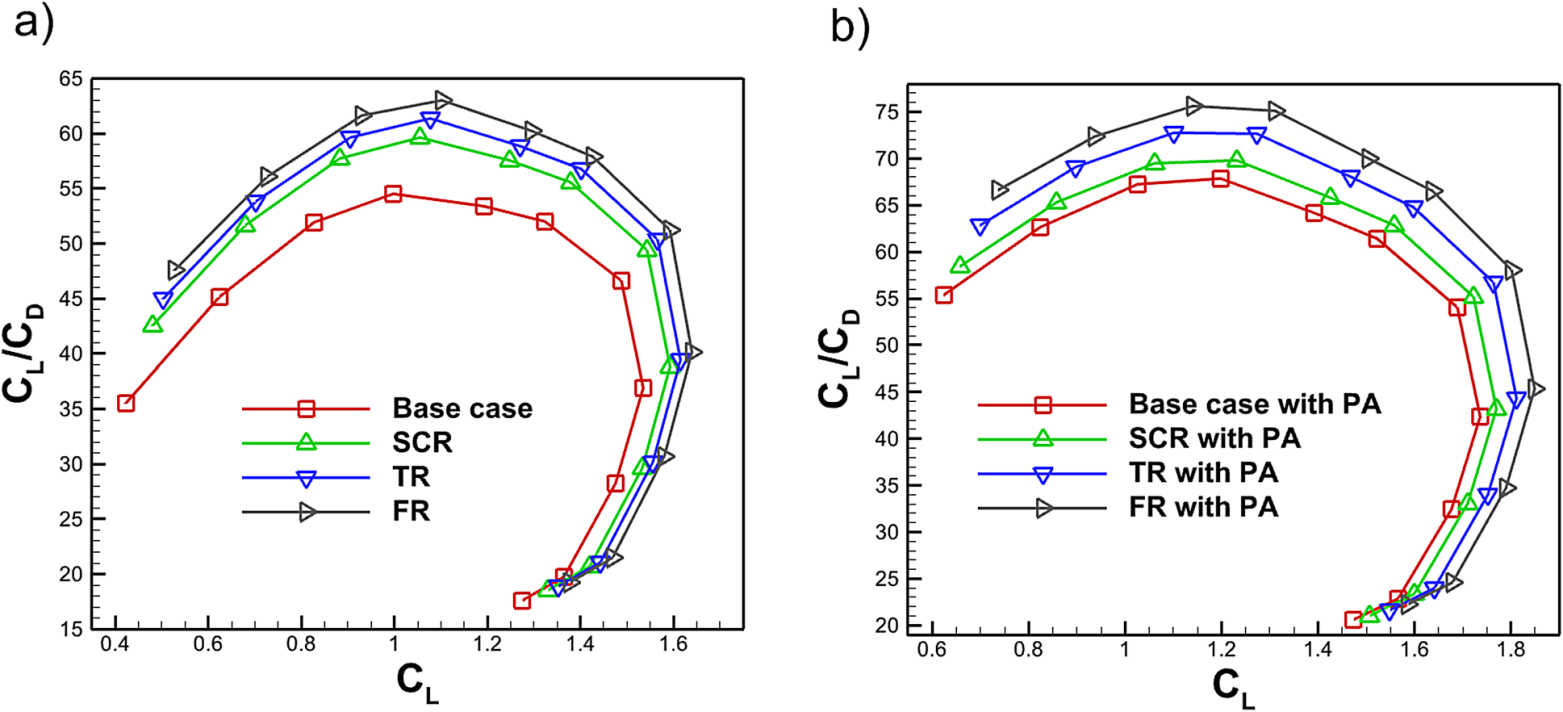
Variation of $C_L/C_D$ with $C_L$ for the base case and riblet equipped airfoil **a** without PA **b** with PA.

The addition of riblets significantly lowers the $C_D$ at angles below stall with a small increase in the lift thus improving the $C_L$/$C_D$ of the airfoil. Figure 19a, b shows different geometries of riblets enhance the $C_L$ therefore improving the $C_L$/$C_D$. For example, once $C_L$ surpasses 1.53, the fillet riblet consistently demonstrates a higher $C_L$/$C_D$ ratio compared to the baseline case, as shown in Fig. 19a. Furthermore, Fig. 19b highlights that at a maximum $C_L$ of 1.84, the combination of a fillet riblet with a plasma actuator yields an improvement of approximately 22.91% in the $C_L$/$C_D$ ratio. As shown in Fig. 19 the observed results shows excellent agreement with the benchmark data and previous investigations^82,83^.

**Fig. 20:**
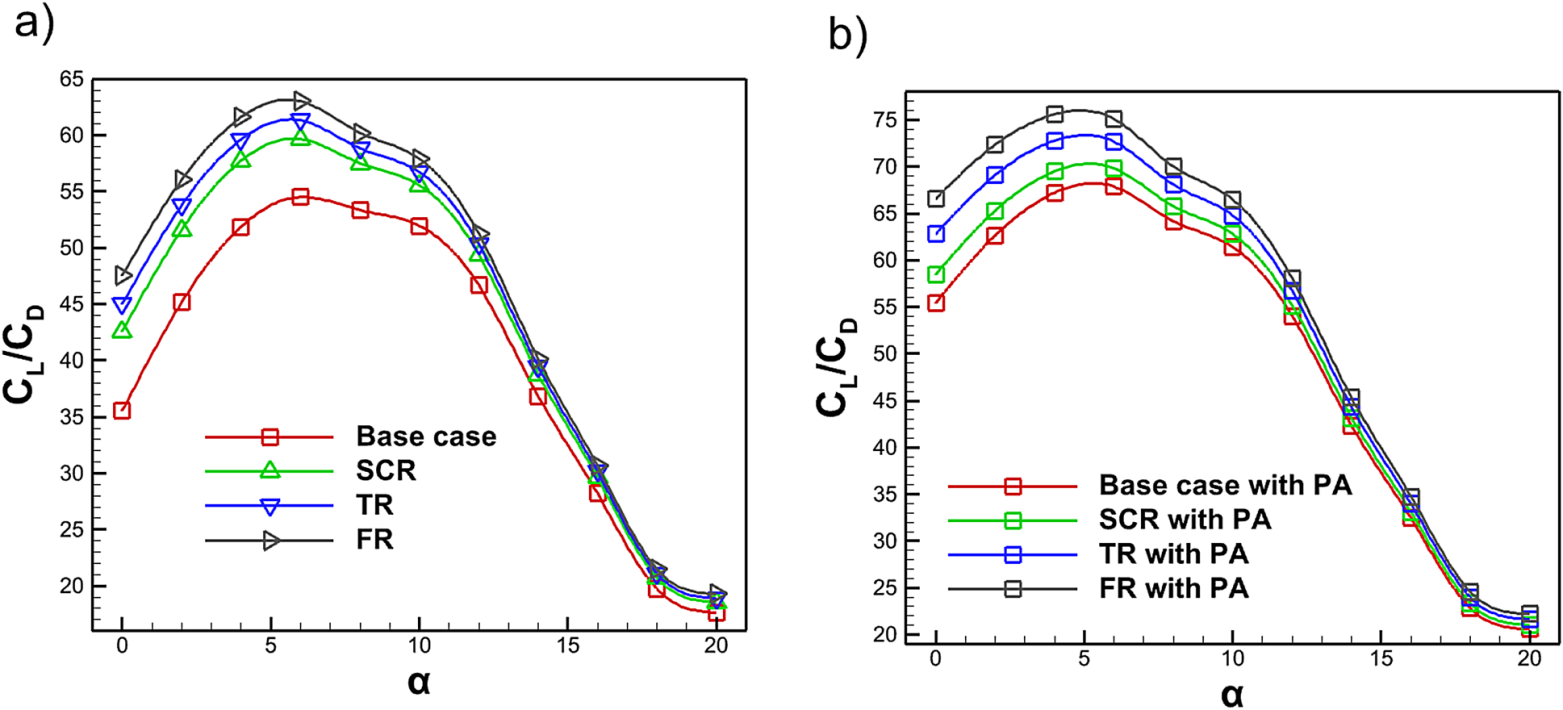
Variation of $C_L/C_D$ with AOA for the base case and riblet equipped airfoil **a** without PA **b** with PA.

Figure 20 presents the variation of the $C_L$/$C_D$ as a function of AOA. As shown in Fig. 20a, riblet geometries yield higher $C_L$/$C_D$ values at lower angles of attack. The maximum $C_L$/$C_D$ increases by 11.27%, 14.95% and 18.72% for the semi-circular, triangular and fillet riblets, respectively. However, the presence of riblets slightly alters the airfoil camber which can reduce $C_L$/$C_D$ at certain conditions. In Fig. 20b, when a plasma actuator is incorporated with riblets, all cases exhibit further enhancement of $C_L$/$C_D$ at lower AOA. The maximum improvements are 29.55% for the baseline, 33.95% for the semi-circular riblet, 40.29% for the triangular riblet and 45.71% for the fillet riblet with a plasma actuator, the latter providing the most significant benefit. As shown in Fig. 20 the observed results shows excellent agreement with the benchmark data and previous investigations^82,83^. Across all configurations $C_L$/$C_D$ rises sharply with AOA, reaches a peak and then gradually decreases. At higher AOA, the $C_L$/$C_D$ ratios converge toward values similar to those of the baseline case. Integrating riblets near the leading edge suction side with plasma actuators can substantially enhance aerodynamic performance while mitigating riblet limitations even at high Reynolds numbers^53,54^.

### Performance of ANN and SVM model

Artificial neural network and support vector machine regression models were constructed based on CFD generated data obtained from various airfoil configurations. The correlation coefficient and mean squared error were used to evaluate their prediction ability. The data were partitioned such that 70% was allocated for training while the remaining 30% was equally split between validation and testing. The ANN was trained using the SCG method while a fine Gaussian SVM was employed to optimize learning efficiency and prediction accuracy. Input parameters included the angle of attack, Reynolds number, plasma actuator frequency, plasma actuator location and total width of riblets with $C_L$ and $C_D$ as outputs. Configurations considered included the base case and three riblet types with an AC-DBD plasma actuator.

**Fig. 21:**
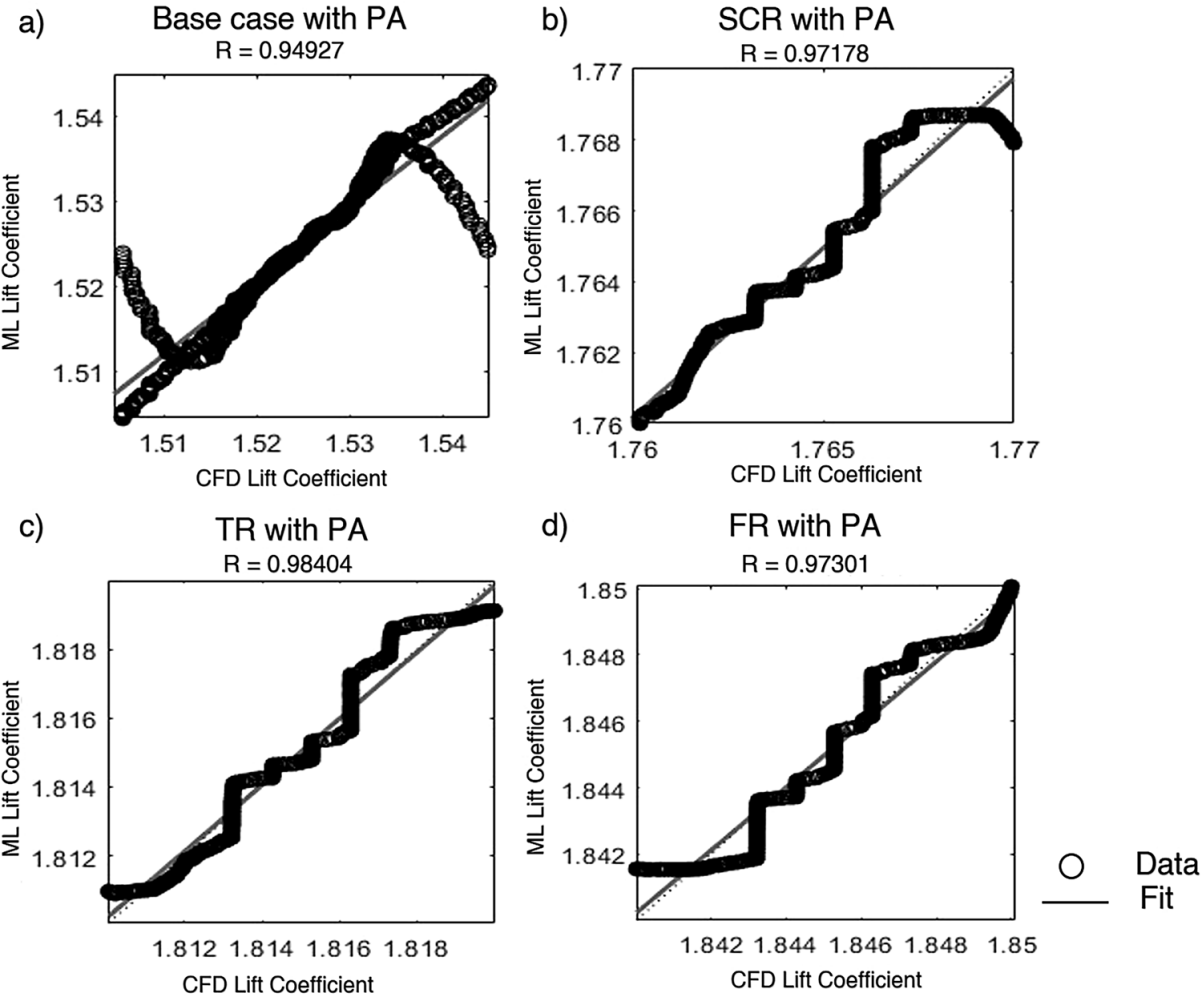
Lift coefficient regression graphs for different riblet with PA using the scaled conjugate gradient method.

Figure 21 presents a comparison between the CFD computed lift coefficient and the ANN predictions obtained using the SCG algorithm for all plasma actuated cases. The base case with PA in Fig. 21a exhibited a correlation coefficient of $R = 0.94927$ and an MSE of $7.5723 \times 10^{-6}$. The semi-circular riblet with PA in Fig. 21b achieved $R = 0.97178$ and MSE = $4.3632 \times 10^{-6}$, while the triangular riblet with PA in Fig. 21c demonstrated the highest accuracy with $R = 0.98404$ and MSE = $2.3076 \times 10^{-6}$. The bio inspired fillet riblet with PA in Fig. 21d showed $R = 0.97301$ and MSE = $3.9340 \times 10^{-6}$. These results confirm the effectiveness of the SCG algorithm in capturing complex aerodynamic trends promoted by riblets and plasma actuation. The triangular riblet configuration offers the highest predictive accuracy and making strong impact on the enhancement of both aerodynamic performance and model reliability..

**Fig. 22:**
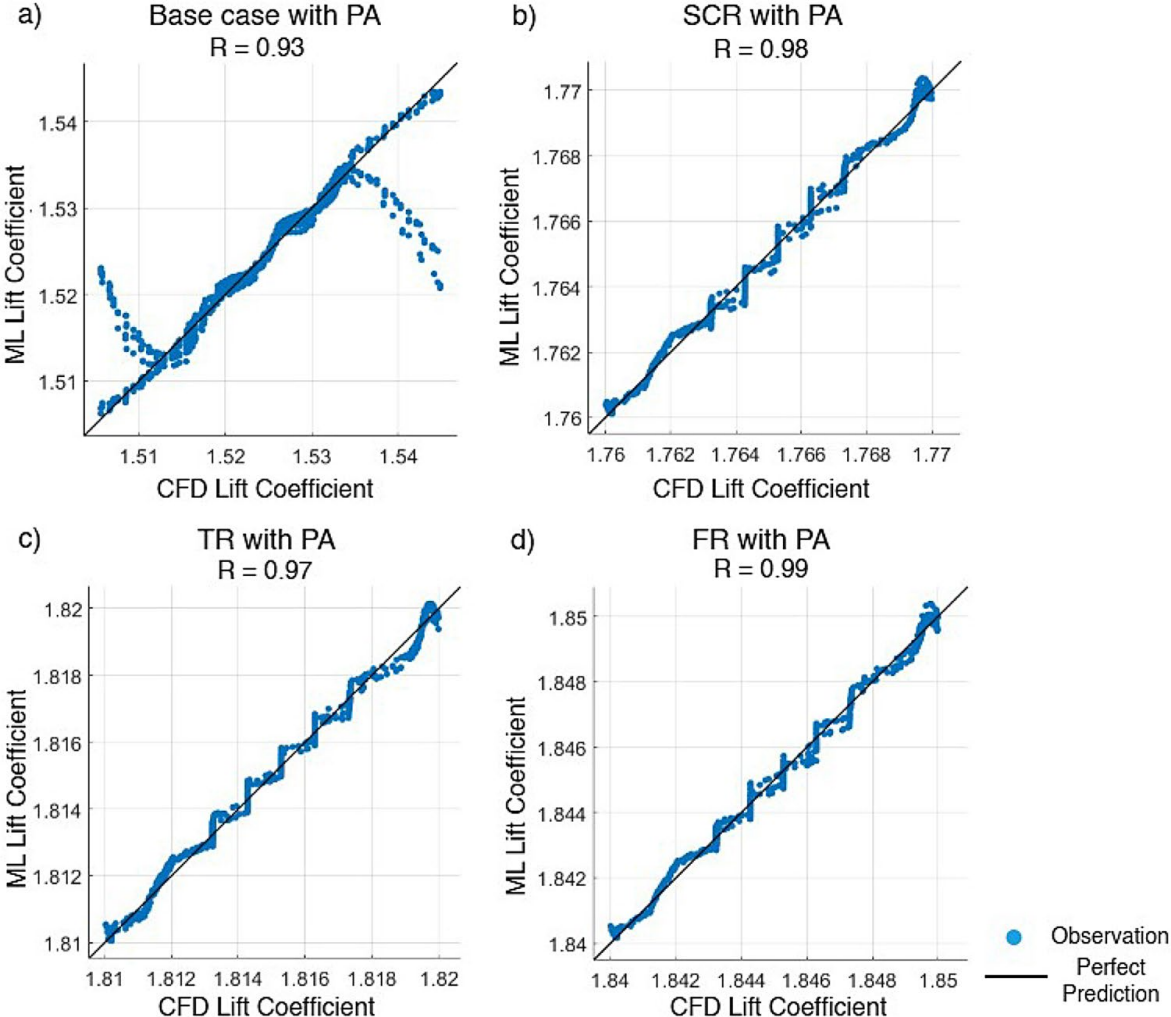
Lift coefficient regression graphs for different riblet with PA using the support vector machine method.

Figure 22 presents the comparison of the $C_L$ values predicted by the SVM model and those obtained from CFD simulations for the four different configurations tested with plasma actuators. For the baseline configuration with plasma actuation shown in Fig. 22a the ANN model achieved a correlation coefficient of $R=0.93$ with a mean squared error of $1.2283\times 10^{-5}$ which indicates a reasonably strong agreement but comparatively lower accuracy. The semi-circular riblet with plasma actuator of Fig. 22b reached the higher correlation $R = 0.98$ with a significantly lower MSE of $6.9946 \times 10^{-8}$ that underlines improved predictive precision caused by the riblet influence on near wall flow structures. The triangular riblet with PA configuration in Fig. 22c achieved $R = 0.97$ and MSE of $7.3995 \times 10^{-8}$ by demonstrating strong correlation and low prediction error. Finally the fillet riblet with plasma actuator shown in Fig. 22d reached the highest correlation $R = 0.99$ with an MSE of $8.6266 \times 10^{-8}$ by indicating the most accurate SVM prediction among the tested configurations. Overall, that integrating riblet geometries with plasma actuation on airfoil enhances flow smoothness, suppresses turbulence and improves the ability of the SVM surrogate model to accurately predict $C_L$. The results emphasize that riblet design significantly influences aerodynamic predictability with bio inspired fillet and semi-circular riblets offering the most substantial improvements in model accuracy for advanced flow control studies.

**Fig. 23:**
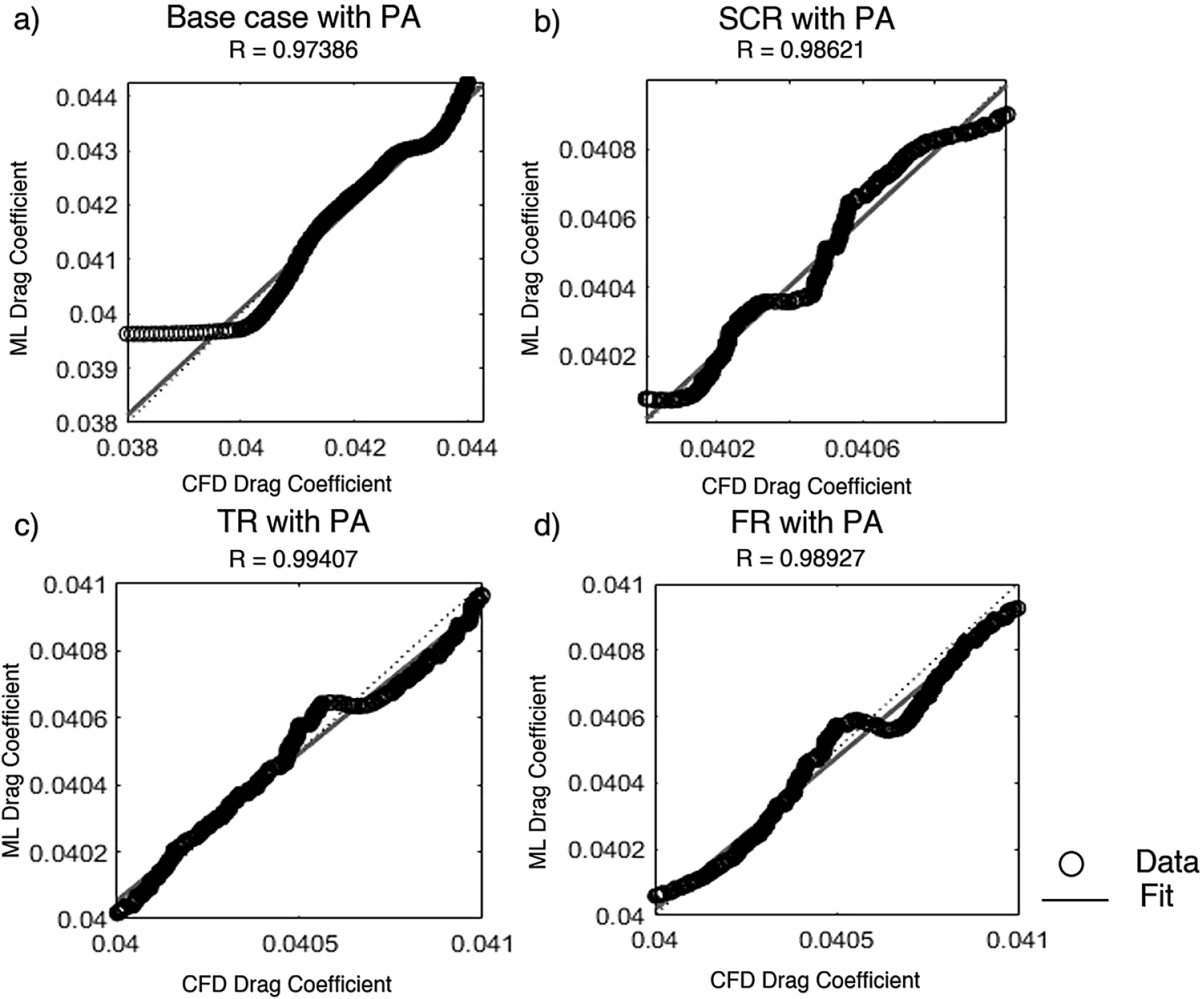
Drag coefficient regression graphs for different riblet with PA using the support vector machine method.

Figure 23 shows the regression results for the $C_D$ predicted by the SCG trained ANN model. The correlation coefficient values demonstrate excellent agreement between the ML predictions and CFD results across all cases. The base case with plasma actuator shown in Figure 23a achieved $R = 0.97386$ with an MSE of $1.0714 \times 10^{-7}$. The semi circular riblet with plasma actuator in Fig. 23b yielded $R = 0.98621$ and the lowest MSE of $2.2620 \times 10^{-9}$ this underlines increased predictive accuracy as a result of flow modifications owing to riblets. On the other hand the triangular riblet with plasma actuator configuration shown in Fig.23c achieved the highest correlation $R = 0.99407$ with MSE of $2.4539\times 10^{-9}$ these low values designate very strong model performance and highly accurate $C_D$ prediction. The fillet riblet with PA arrangement in Fig. 23d finally obtained $R = 0.98927$ with MSE $= 2.5863 \times 10^{-9}$ and demonstrated high prediction fidelity. These findings assure that the use of riblets with plasma actuators enhances flow predictability as a result of which generalization capability of the SCG trained ML model improves and the $C_D$ is predicted accurately on diverse aerodynamic surface textures.

**Fig. 24:**
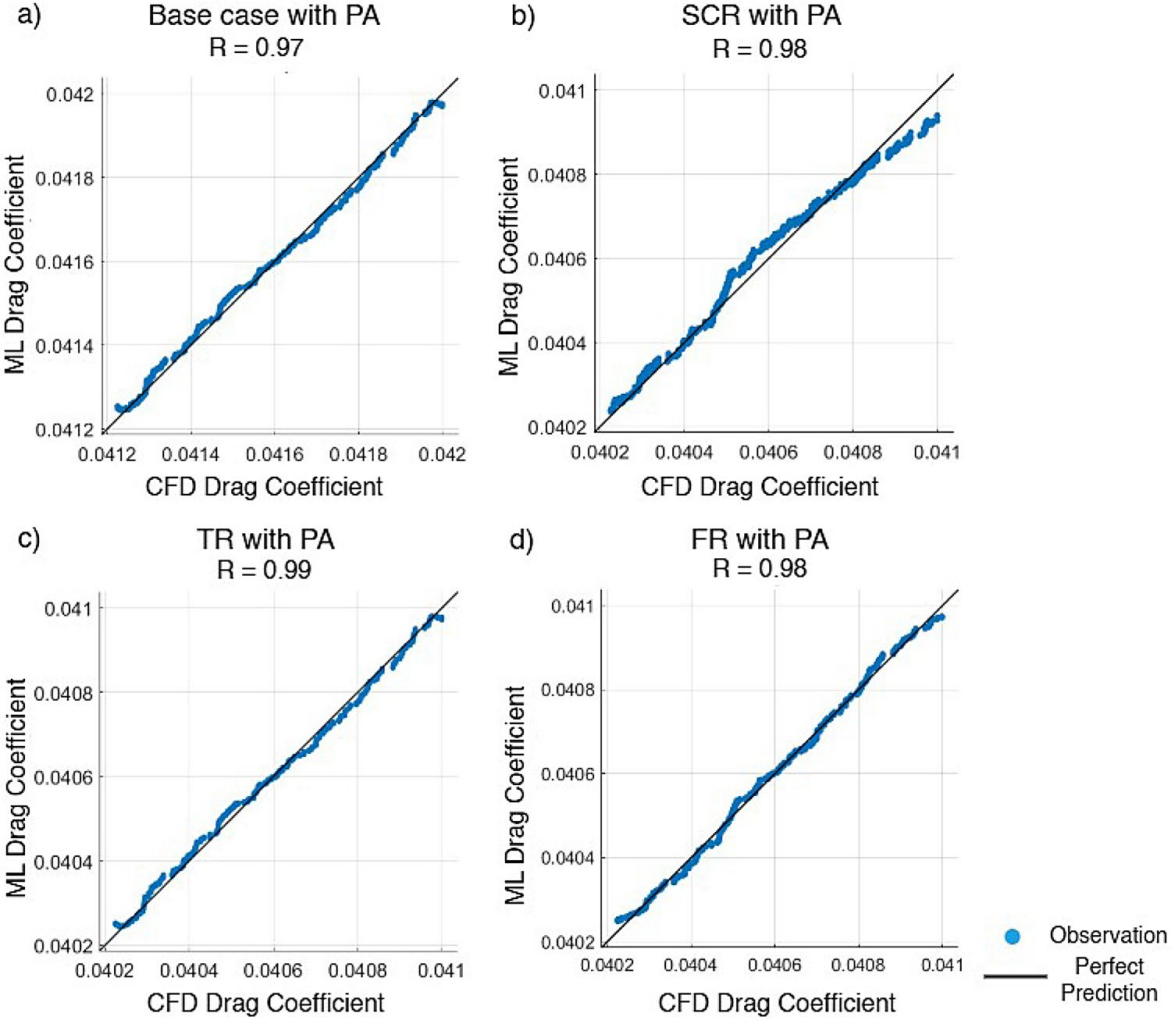
Regression plots of drag coefficient for various riblets with PA using the support vector machine technique.

Figure 24 presents a comparison of $C_D$ predicted using the SVM model with corresponding values determined from CFD simulations for four different riblets geometries with plasma actuators. The *R* values in all of the cases confirm a good linear agreement between ML predicted and CFD computed $C_D$. The plasma actuator on the base case shown in Fig. 24 presented a correlation of $R = 0.97$ and a MSE of $2.3578 \times 10^{-10}$. Figure 24b presents the semi-circular riblet with plasma actuator where $R = 0.98$ is reached with MSE of $7.7538 \times 10^{-10}$. For the triangular riblet with PA configuration presented in Fig. 24c, the highest correlation of $R = 0.99$ with a MSE of $2.3718 \times 10^{-10}$ was realized which shows very good predictive performance. The fillet riblet with plasma actuator in Fig. 24d also presented $R = 0.98$ and the least value of MSE $1.3065 \times 10^{-10}$. It is found that the developed SVM based ML model is highly reliable in predicting aerodynamic behavior for different surface modifications. In addition very small MSE value for all four models confirms its reliability and capability in the estimation of the drag characteristics. In summary, the results confirm that combining riblet geometries with plasma actuation may have several potentials in yielding better aerodynamic performances. Finally, this study also underlines SVM as a strong surrogate modeling methodology within the framework of aerodynamic optimization and flow control studies.

## Conclusions

This study presented a CFD-ML investigation of hybrid flow control on a NACA4412 airfoil at a Reynolds number of $3.1 \times 10^6$ by integrating bio inspired riblet geometries with AC-DBD plasma actuators and predictive machine learning models. The most important findings are:

- A distinct synergy between riblets and plasma actuation was noted. Riblets reduced skin friction drag and slightly increased lift that had a tendency to intensify separation at higher angles of attack. Plasma actuators offset this weakness by energizing the boundary layer which delays separation and improves flow stability.
- Among the configurations evaluated the bio-inspired fillet riblet (FR) combined with AC-DBD actuation exhibited the greatest aerodynamic improvement by achieving up to 20.38% increase in lift and 6.20% reduction in drag. This resulted in a maximum enhancement of 45.71% in the $C_L/C_D$ ratio with particularly strong performance in the $0^\circ$-$14^\circ$ angle of attack range.
- Machine learning models provided highly accurate aerodynamic predictions. The ANN trained with the scaled conjugate gradient algorithm has resulted in strong correlations ($R > 0.98$) and low MSE values, especially for triangular riblet with plasma cases which confirms its reliability in capturing complex flow behavior. Similar performance in terms of excellent agreement with CFD was obtained by SVM regression across all configurations for $R \approx 0.99$ for both lift and drag.
- The bio inspiration assisted FR with AC-DBD produced the most effective total aerodynamic performance by making it very suitable for a wide range of tasks where a distinct flight range or fuel economy is required. The approach of using CFD-ML in this study can mitigate over-dependence on rigorous computations in designing advanced hybrid flow control systems.

## Data Availability

Data available on request from the authors
